# S1‐Leitlinie Schweißdrüsenkarzinom

**DOI:** 10.1111/ddg.70107

**Published:** 2026-09-08

**Authors:** Mirjana Ziemer, Michael Erdmann, Abbas Agaimy, Jürgen C. Becker, Almut Böer‐Auer, Doris Helbig, Cornelia Erfurt‐Berge, Alexander Frisman, Conrad Hempel, Ulrike Leiter, Friedegund Meier, Thomas Mentzel, Lara Racz, Silke Redler, Stefan Schliep, Clemens Seidel, Alpaslan Tasdogan, Jochen Utikal, Carsten Weishaupt, Selma Ugurel

**Affiliations:** ^1^ Klinik für Dermatologie, Venerologie und Allergologie Hauttumorzentrum Leipzig, Mitteldeutsches Krebszentrum (CCCG), Universitätsklinikum Leipzig Leipzig Deutschland; ^2^ Hautklinik, Uniklinikum Erlangen, Comprehensive Cancer Center (CCC) Erlangen‐EMN Bavarian Center for Cancer Research (BZKF), Friedrich‐Alexander Universität Erlangen‐Nürnberg Erlangen Deutschland; ^3^ Institut für Pathologie, Uniklinikum Erlangen, Friedrich‐Alexander Universität Erlangen‐Nürnberg Erlangen Deutschland; ^4^ Translationale Hautkrebsforschung, Deutsches Konsortium für Translationale Krebsforschung (DKTK), Universitätsmedizin Essen und Deutsches Krebsforschungszentrum (DKFZ) Heidelberg Deutschland; ^5^ Dermatologikum Hamburg GmbH Hamburg Deutschland; ^6^ Klinik und Poliklinik für Dermatologie und Venerologie Uniklinik Köln Köln Deutschland; ^7^ Klinik für Strahlentherapie Universitätsklinikum Leipzig Leipzig Deutschland; ^8^ Universitäts‐Hautklinik Universitätsklinikum Tübingen Tübingen Deutschland; ^9^ Klinik und Poliklinik für Dermatologie Nationales Centrum für Tumorerkrankungen/Universitäts KrebsCentrum Dresden Dresden Deutschland; ^10^ Kressbronn Baden‐Württemberg Deutschland; ^11^ Institut für Humangenetik, Medizinische Fakultät und Universitätsklinikum Düsseldorf, Heinrich‐Heine‐Universität Düsseldorf Düsseldorf Deutschland; ^12^ Klinik für Dermatologie, Venerologie und Allergologie Universitätsmedizin Essen Essen Deutschland; ^13^ Kooperationseinheit Dermatoonkologie des Deutschen Krebsforschungszentrum Heidelberg (DKFZ) und der Klinik für Dermatologie, Venerologie und Allergologie Universitätsmedizin Mannheim, Ruprecht‐Karls‐Universität Heidelberg, Mannheim und DKFZ Hector Krebsinstitut an der Universitätsmedizin Mannheim Mannheim Deutschland; ^14^ Klinik für Hautkrankheiten, Allgemeine Dermatologie und Venerologie Universitätsklinikum Münster Münster Deutschland; ^15^ Universitätsklinik für Dermatologie, Venerologie und Allergologie Klinikum Bielefeld Rosenhöhe, Medizinische Fakultät und Universitätsklinikum OWL der Universität Bielefeld Bielefeld Deutschland

**Keywords:** classification, diagnosis, guideline, prognosis, treatment, sweat gland carcinomas

## Abstract

Die aktuelle Einteilung der Schweißdrüsenkarzinome erfolgt nach histomorphologischen Charakteristika und unterscheidet mehr als 20 Entitäten. Es sind meist Patienten höheren, bei einigen Subtypen aber auch mittleren und jüngeren, Lebensalters betroffen. Die Mehrheit der Tumoren entsteht de novo. Schweißdrüsenkarzinome besitzen eine unspezifische Klinik. Die Tumoren sind meist im Kopf‐Hals‐Bereich und an den Extremitäten lokalisiert. Eine Ausnahme bildet mit anogenitaler Prädilektion das extramammäre Paget‐Karzinom. Schweißdrüsenkarzinome, die in der Achselhöhle auftreten, können eine histomorphologische Herausforderung in der Abgrenzung zum metastasierten oder primären Mammakarzinom darstellen. Die Diagnose erfolgt über eine (Probe‐)exzision und Histopathologie. Die histopathologische Subdifferenzierung ist hierbei für eine exakte Diagnosestellung von essentieller Bedeutung. Eine Metastasierung erfolgt zunächst lokal fortschreitend (per continuitatem), später mit Befall der regionären Lymphknoten und entfernter Organe. Die Therapie der ersten Wahl ist die vollständige chirurgische Exzision unter allseitiger histologischer Schnittrandkontrolle (mikroskopisch kontrollierte Chirurgie). Alternativ kann eine weite lokale Exzision durchgeführt werden mit einem Sicherheitsabstand. Eine postoperative adjuvante Strahlentherapie wird bei Hochrisikotumoren empfohlen. Die Datenlage für medikamentöse Therapien fortgeschrittener Tumoren ist insgesamt schwach, am besten noch für Chemotherapien unter Einsatz von Platinderivaten. Des Weiteren sind zielgerichtete Therapien möglich, zum Beispiel mit HER2/neu‐ oder EGFR‐Inhibitoren, eventuell in Kombination mit Chemotherapie. Für die Nachsorge wird ein risikoadaptiertes Schema empfohlen.

## EPIDEMIOLOGIE UND ÄTIOPATHOGENESE

*Kapitelautoren*: Conrad Hempel, Mirjana Ziemer, Michael Erdmann

### Epidemiologie

Schweißdrüsenkarzinome sind seltene Neoplasien der Haut.^1^ Sie stellen eine Gruppe verschiedener Entitäten dar, denen der Ursprung aus den apokrinen oder ekkrinen Schweißdrüsen der Haut gemeinsam ist. Insgesamt machen Schweißdrüsenkarzinome nur etwa 0,05 % aller malignen Hauttumoren aus.^2^ Sie stellen jedoch – nach dem Basalzellkarzinom als follikulär differenziertem Tumor – innerhalb der Adnexkarzinome den Hauptanteil dar (Inzidenz 5,1 pro 10^6^).^1^, ^3^ Die Inzidenz ist steigend.^2^, ^4^ Männer und Frauen sind über alle Entitäten betrachtet nicht signifikant unterschiedlich häufig betroffen. Eine Besonderheit stellt bei einigen Entitäten – wie zum Beispiel dem digitalen papillären Adenokarzinom – das jüngere Alter der Patienten dar. Bedeutsam ist auch die Topografie der Tumoren, da in hautnahen Organen wie den Speicheldrüsen und auch in der Mamma morphologisch sehr ähnliche, aber biologisch andersartige Tumoren vorkommen.

Die sichere Unterscheidung der einzelnen Schweißdrüsentumorentitäten hat eine prognostische Bedeutung. Während einige Entitäten, wie zum Beispiel das mikrozystische Adnexkarzinom, nach gesicherter vollständiger Exzision eine sehr gute Prognose aufweisen, zeigen andere schlechtere Überlebensraten und müssen entsprechend differenziert behandelt und nachgesorgt werden.

### Ätiologie

Die meisten Adnexkarzinome entstehen *de novo* und sporadisch. Seltener ist eine maligne Transformation benigner Tumoren.^3^ Als mögliche ätiopathogenetische Triggerfaktoren kommen UV‐Strahlung, eine vorangegangene Strahlentherapie, das Vorliegen einer Immunsuppression, aber ursächlich auch genetische Erkrankungen in Betracht.^5^ Insbesondere für das Porokarzinom und das Hidradenokarzinom verdichten sich die Daten, die in der Tumorgenese onkogene Treiber in den PI3K/AKT (Phosphoinositid‐3‐Kinase/Proteinkinase B)‐ und MAPK (mitogen‐activated protein kinase)‐Signalwegen nahelegen. Zudem konnte bei Porokarzinomen eine hohe Tumormutationslast nachgewiesen werden, die der UV‐Exposition zugesprochen wird.^6^, ^7^ Für das digitale papilläre Adenokarzinom ist inzwischen eine HPV‐assoziierte Tumorentwicklung belegt.^8^ Für einige Tumoren wurden spezifische genetische Veränderungen nachgewiesen, wie zum Beispiel die *MYB::MYBL1*‐Fusion beim adenoid‐zystischen Karzinom und die *YAP1::NUTM1‐, YAP1::MAML2‐* beziehungsweise *YAP1::WWTR1*‐Fusionen beim Porokarzinom. Eine tumorassoziierte genetische Erkrankung mit gehäuftem Auftreten von Schweißdrüsenkarzinomen ist das Brooke‐Spiegler‐Syndrom.^9^

## KLASSIFIKATION

*Kapitelautoren*: Mirjana Ziemer, Almut Böer‐Auer

Schweißdrüsenkarzinome imitieren histologisch Gangstrukturen und/oder sekretorische Endstücke apokriner oder ekkriner Drüsen.

Apokrine Drüsen (*glandulae sudoriferae apocrinae*) sind Teil der embryonalen apokrin‐follikulär‐sebaziösen Einheit und münden oberhalb der Talgdrüsen in den Haarfollikel (gelegentlich auch direkt in die Epidermis). Ihr Verteilungsmuster lässt sich leichter darüber merken, dass sie vor allem in Hautregionen lokalisiert sind, die mit dem Buchstaben „A“ anfangen: Achselhöhlen (axillär), Brustwarzen (areolär), anogenital, Gesicht (Antlitz), dort insbesondere Augenlider. Vereinzelt finden sie sich auch in der Regio pubica sowie periumbilikal. Die Ceruminaldrüsen des äußeren Gehörgangs und die Moll‐Drüsen der Augenlidkanten werden als modifizierte apokrine Drüsen betrachtet.

Ekkrine Drüsen (glandulae sudoriferae eccrinae) haben keinen Bezug zum Haarfollikel und münden über ein Akrosyringium direkt in die Epidermis.

Die Zuordnung der Tumoren in ekkrine und apokrine Differenzierung ist jedoch nicht immer möglich, da die Gangstrukturen beider Drüsen identisch aufgebaut sind und separierende Merkmale wie apokrine Sekretion oder Assoziation mit embryologisch verwandten Geweben wie Haarfollikel‐ oder Talgdrüsendifferenzierung oft fehlen. Zudem sind von vielen Entitäten sowohl apokrine als auch ekkrine Varianten beschrieben. Nicht zuletzt hat die Abgrenzung derzeit keine therapeutischen Konsequenzen.

Erste Einteilungen der Schweißdrüsenkarzinome aus den 1950er Jahren waren primär auf partikuläre histomorphologische Charakteristika gestützt. So unterteilten Stout und Cooley die Tumoren in entweder adenoid‐zystische, muzinöse oder duktale Karzinome.^10^ Im Jahr 1968 haben Berg und McDevitt auf Basis einer über 100 Fälle einschließenden Serie eine erweiterte Klassifikation vorgestellt.^11^

Eine weitere Unterteilungsmöglichkeit besteht nach dem Metastasierungsrisiko in niedrig‐ und hochmalige Tumoren. Niedrigrisiko‐Tumoren können bei fehlendem operativem Sicherheitsabstand oder nicht mikroskopisch kontrolliert erfolgter Chirurgie zwar durchaus ein erhöhtes Rezidivrisiko aufweisen, metastasieren aber sehr selten, und dann in der Regel in die lokoregionalen Lymphknoten.

Trotz ihrer Seltenheit hat sich gerade bei den Schweißdrüsentumoren eine Vielzahl, zum Teil nur schwer untereinander abgrenzbarer Subtypen etabliert. Das gilt sowohl für die benignen Schweißdrüsentumoren wie auch für deren maligne Pendants. Eine systematische Klassifikation liegt – abgesehen von der histopathologischen WHO‐Einteilung – bislang nicht vor, was eine klare Gesamtbetrachtung der Gruppe erschwert. Es sind mehr als 20 maligne Entitäten beschrieben, von denen die wichtigsten in Tabelle 1 aufgeführt sind.^12^ In vielen Fällen stellen die Subtypen der Schweißdrüsenkarzinome maligne Varianten ihrer gutartigen Pendants dar. Eine zusätzliche Schwierigkeit besteht darin, dass in anderen hautnahen Organen wie zum Beispiel den Speicheldrüsen und auch in der Mamma morphologisch sehr ähnliche Tumoren mit gleicher oder auch divergenter Terminologie aber teilweise sehr unterschiedlicher Prognose vorkommen können, so dass je nach Topographie zunächst sichergestellt werden muss, ob tatsächlich ein von der Haut ausgehender Adnextumor vorliegt, oder andere Organe als Ursprung in Betracht gezogen werden müssen. Unterstützend kann dabei die Immunphänotypisierung sein.

Die Verschlüsselung der Schweißdrüsenkarzinome im ICD‐O umfasst im Kapitel der *Neoplasien der Haut und der Hautanhangsgebilde* die Ziffern 839‐842 (zum Beispiel 8400/3 Schweißdrüsenkarzinom).

## DIAGNOSTIK

*Kapitelautoren*: Almut Böer‐Auer, Mirjana Ziemer, Thomas Mentzel, Abbas Agaimy, Doris Helbig, Stefan Schliep

### Klinik

Schweißdrüsenkarzinome treten, von einigen Ausnahmen abgesehen, meist im fortgeschrittenen Lebensalter in der Kopf‐Hals‐Region auf.^1^ Sie haben keine spezifischen klinischen Merkmale. Häufig treten sie in Form von erythematös‐livide Knötchen und Knoten in Erscheinung. Anders als ihre benignen Pendants neigen Schweißdrüsenkarzinome nicht selten zur Ulzeration. Verdachtsdiagnosen umfassen gutartige Zysten, Basalzellkarzinome oder Plattenepithelkarzinome der Haut. Ausnahmen sind das extramammäre Paget‐Karzinom und das Siegelringzell‐Karzinom der Haut, bei welchen nicht selten klinisch die initiale Fehldiagnose einer entzündlichen Dermatose gestellt wird. Patienten mit Schweißdrüsenkarzinomen sind im Regelfall asymptomatisch.^13^ Bei der klinischen Untersuchung und Anamnese sollte auf Risikofaktoren wie UV‐Strahlung, vorangegangene Strahlentherapie, Immunsuppression, HPV‐Infektion, genetische Aberrationen und genetische Syndrome wie das Brooke‐Spiegler‐Syndrom als auch hämatoproliferative Erkrankungen (insbeasondere chronisch lymphatische Leukämie) geachtet werden.

### Allgemeine Histopathologie

Eine sichere histomorphologische Zuordnung zu apokriner oder ekkriner Differenzierung ist im Fall von Schweißdrüsentumoren aufgrund der Gemeinsamkeiten nicht immer zuverlässig möglich. Die Drüsenausführungsgänge sind bei ekkrinen und apokrinen Drüsen identisch aufgebaut. Das zweireihige Gangepithel besteht aus kleinen, eosinophilen Zellen, die lumenwärts mit einer eosinophilen Kutikula abschließen. Die peripheren Zellen sind basophiler und kubisch geformt mit runden Zellkernen. Eine myoepitheliale äußere Zellschicht fehlt. Die sekretorischen Endstücke bestehen aus einer einreihigen Schicht kubischer oder kolumnarer Zellen umgeben von myoepithelialen Zellen.

Kriterien apokrin differenzierter Tumoren umfassen folgende:

− Nachweis einer sogenannten „Dekapitationssekretion“ (lumennahe Zytoplasma‐Abschnitte werden abgeschnürt und als Sekret in das Drüsenlumen abgegeben),
− elongierte und verzweigte tubuläre Strukturen,
− Verbindung des Tumors zu einem Infundibulum eines Haarfollikels,
− Tumoranteile mit Merkmalen der follikulären und/oder sebaziösen Differenzierung (insbesondere in benignen Adnextumoren oder in Schweißdrüsenkarzinomen, die in ihrem benignen Pendant entstehen),
− besondere zytologische Merkmale: reichliches granulär‐eosinophiles Zytoplasma mit isomorphen zentralen Kernen und erkennbaren Nukleolen.

Eine ekkrin‐sekretorische Differenzierung ist hingegen charakterisiert durch:

− ein flaches sekretorisches Epithel ohne morphologisch Sekretion in das Lumen.

Apokrine wie auch ekkrine sekretorische Endstücke zeigen eine periphere myoepitheliale Schicht, so dass Schweißdrüsentumoren eine prominente myoepitheliale Differenzierung aufweisen können. Der Nachweis eines solchen biphasischen (epithelial‐myoepithelialen) Aufbaus kann auch bei Karzinomen vorkommen und kann daher nicht als grundsätzlicher Benignitätsmarker verwendet werden.

Generell weisen Schweißdrüsenkarzinome im Gegensatz zu ihren (soweit vorhanden) gutartigen Pendants histopathologisch Zellatypien, Kernpleomorphien, dicht gedrängt stehende und überlappende Zellkerne (crowding) und zahlreiche Mitosen, Zellnekrosen sowie ein infiltrierendes Tumorwachstum auf.

Es sei darauf hingewiesen, dass die Histopathologie nicht in allen Fällen ein Prädiktor für den klinischen Verlauf ist, da sich ein Teil der Tumoren trotz eines blanden histopathologischen Musters aggressiv verhalten kann.

#### Immunphänotypisierung und molekularpathologische Marker

Das Drüsenepithel sowohl der apokrinen als auch der ekkrinen Drüsen kann immunhistochemisch (abgesehen von Panzytokeratinmarkern) mit Zytokeratin (CK) 7, CK 8, CK 18 und *gross cystic disease fluid protein 15* (GCDFP‐15, ein sonst für Mammakarzinome typischer Marker) dargestellt werden. Die Drüsenlumina‐nahen Zellen färben sich mit Antikörpern gegen CEA (carcinoembryonales Antigen) und EMA (epitheliales Membranantigen). Diese Färbungen sind jedoch nicht spezifisch. Die sekretorischen Endstücke der apokrinen und ekkrinen Drüsen sind umgeben von einer äußeren flachen myoepithelialen Zellschicht, die sich mit den Markern Glattmuskelaktin, p63 oder Calponin darstellen lässt. So lässt sich mit p63 immunohistochemisch das myoepitheliale Muster bei allen digitalen papillären Adenokarzinomen nachweisen, beim Porokarzinom und klarzelligen Hidradenokarzinom hingegen weist die p63 Färbung ein diffuses Muster auf. In Schweißdrüsenkarzinomen können die Expressionsmuster jedoch weniger konsistent sein als in den benignen Pendants oder in regulären Drüsen, wobei auch metaplastische Phänomene, wie squamoide oder muzinöse Metaplasie, zu berücksichtigen sind.^15^

Schweißdrüsenkarzinome exprimieren häufig den EGF‐Rezeptor (epidermal growth factor receptor, EGF‐R). Dies kann differenzialdiagnostisch zur Abgrenzung von Metastasen eines Mammakarzinoms hilfreich sein, da eine Expression von EGF‐R beim Mammakarzinom deutlich seltener vorliegt. Zu berücksichtigen ist jedoch seine unzureichende Spezifität, insbesondere in der Abgrenzung zu anderen Tumoren mit squamöser Differenzierung. Die Expression von Östrogen‐ und/oder Progesteronrezeptoren unterscheidet hingegen nicht sicher zwischen Schweißdrüsenkarzinomen und Mammakarzinommetastasen.^14^ Insbesondere bei muzinösen Schweißdrüsenkarzinomen zeigt sich ein identisches Profil wie bei muzinösen Mammakarzinomen mit starker homogener Expression von Östrogen‐ oder Progesteronrezeptoren und häufig von Synaptophysin. Der Nachweis von Östrogen‐ und/oder Progesteronrezeptoren spielt gegebenenfalls für einen möglichen antihormonellen Therapieansatz mit Tamoxifen eine Rolle.^15^ Zudem ist eine globale Expression von Androgenrezeptoren ein reguläres Merkmal apokriner Adenokarzinome. Weiterhin sprechen die Expression von CK 5/6 und p63 eher für ein primäres Schweißdrüsenkarzinom und gegen eine Metastase.

In den letzten Jahren haben sich weitere immunhistochemische und molekulare Marker etabliert, die eine Differenzierung der Schweißdrüsentumoren erleichtern können. So zeigen alle Porokarzinome entweder eine Aktivierung von NUT (nuclear protein of testis) mittels positiver immunhistochemischer Färbung oder ein *YAP1::NUTM1*‐Fusionsgen in der RNA Sequenzierung (yes‐associated protein [YAP] 1). Die MAML2‐Fluoreszenz‐in‐situ‐Hybridisierung (FISH) ist bei allen klarzelligen Hidradenokarzinomen positiv.^15^

Von Interesse – insbesondere in Hinblick auf die Prognose aber auch den therapeutischen Ansatz – ist die PD‐L1‐Expression und der Anteil tumorinfiltrierender CD8‐postiver Lymphozyten.^16^ Diese wurden mittels Immunhistochemie an 83 Adnexkarzinomen untersucht, unter denen sich 60 Schweißdrüsenkarzinome befanden. Von diesen exprimierten 13 % PD‐L1 in ≥ 1 % der Tumorzellen (8/60). Die höchste PD‐L1‐Expression auf Tumorzellen zeigten apokrine Karzinome (40 %, 2/5) und invasive extramammäre Paget‐Karzinome (57 %, 4/7). Immunzellen exprimierten dabei signifikant häufiger PD‐L1 als Tumorzellen. Dichte CD8‐positive tumorinfiltrierende Lymphozyten fanden sich in 43 % der Schweißdrüsenkarzinome. In der univariaten Analyse war die Expression von PD‐L1 durch die Tumorzellen mit der Entwicklung von Metastasen assoziiert (Hazard Ratio [HR] 4,0, 95 %‐Konfidenzintervall [KI] 1,1–15, p = 0,04), nicht hingegen in der multivariaten Analyse (HR 4,1, 95 %‐KI 0,6–29, p = 0,15).

Bei Schweißdrüsenkarzinomen, die in ihren benignen Pendants entstehen, wird der Proliferationsmarker Ki67 im malignen Anteil herdförmig vermehrt exprimiert. Ansonsten zeigt sich der Proliferationsmarker sehr variabel und ist gerade bei hochdifferenzierten Schweißdrüsenkarzinomen nicht immer aussagekräftig erhöht.

### Spezielle Klinik und Histologie von Schweißdrüsenkarzinomen

Die klinisch geläufigsten Varianten werden nachfolgend ausführlicher beleuchtet. Die wichtigen Charakteristika weiterer Schweißdrüsenkarzinome sind in Tabelle 1 aufgeführt.

#### Porokarzinom

Porokarzinome sind die häufigsten Schweißdrüsenkarzinome. Inzidenzen werden in der Literatur mehrheitlich zwischen 0,02 und 0,06 Fällen pro 100 000 Einwohner/Jahr berichtet. Untersuchungen aus England zufolge liegt die Inzidenz mit 1,9 Fällen 100 000 Einwohner/Jahr höher.^2^, ^17^, ^18^, ^19^ Eine Entstehung in den oberen Anteilen der ekkrinen Schweißdrüsenausführungsgänge wird vermutet. In 20–30 % der Porokarzinome kann man ein vorbestehendes Porom nachweisen. Porokarzinome gehören zu den aggressiven Tumoren aus der Gruppe der Schweißdrüsenkarzinome. Die Lokalrezidivrate wird mit etwa 20 % angegeben, wobei diese nach mikroskopisch kontrollierter Chirurgie geringer ausfällt.^20^ Eine Metaanalyse an 453 Patienten aus dem Jahr 2017 ermittelte eine Metastastasierungsrate von 31 %, dabei erfolgte die Metastasierung am häufigsten in Lymphknoten und Lunge.^21^ Einer Publikation aus dem Jahr 1992 folgend wird die Mortalitätsrate nach Lymphknotenmetastasierung mit 67 % angegeben.^22^ Neuere Daten gehen jedoch von niedrigeren Zahlen aus.^23^

Porokarzinome treten überwiegend bei älteren Patienten auf und imponieren klinisch wie Plattenepithelkarzinome. Sie sind mehrheitlich an den unteren Extremitäten und der Kopf‐Hals‐Region lokalisiert.^24^ Bei metastasierenden Verläufen kann sich zwischen dem Primärtumor und der Lymphknotenmetastase ein ausgeprägtes Lymphödem mit zahlreichen epidermotropen Metastasen im Sinne einer lymphatischen Aussaat der Tumorzellen entwickeln.^25^, ^26^, ^27^, ^28^, ^29^

Histologisch zeigen Porokarzinome ein infiltrierendes Wachstum von Epithelverbänden atypischer poroider Zellen mit fokal erkennbarer duktaler Differenzierung und zahlreichen Mitosen. Es können großflächige Tumornekrosen vorliegen, die jedoch nicht als alleiniges Kriterium für Malignität ausreichen, da eine *necrosis en masse* auch bei Poromen vorliegen kann. Die intraepitheliale (in situ) Form des Porokarzinoms wird als malignes Hidroacanthoma simplex bezeichnet und muss histologisch von einem benignen Hidroacanthoma simplex als auch von einem pagetoiden Morbus Bowen und der klonalen seborrhoischen Keratose abgegrenzt werden. Teilweise klarzellige und squamoide Differenzierungen sind nicht selten. In circa 20 % der Fälle wurde eine Kolonisierung mit Melanozyten beschrieben, was sich klinisch mit einer Pigmentierung und somit dem Verdacht auf einen melanozytären Tumor äußern kann.^24^

Viele Porokarzinome zeigen trotz einer geringen Eindringtiefe eine intralymphatische Ausbreitung in der Tumorperipherie. Als prognostisch ungünstige Faktoren gelten eine hohe Mitoserate, eine Tumoreindringtiefe über 7 (–10) mm und der Nachweis einer intralymphatischen Tumorausbreitung.^24^, ^30^, ^31^, ^32^ Als weiteres Kriterium für eine Metastasierung wurde ein infiltratives dermales Muster beschrieben (*scattered pattern of the dermal invasive component* im Gegensatz zum verdrängenden *Pushing*‐Muster).

#### Hidradenokarzinom

Hidradenokarzinome sind neben dem Porokarzinom ebenfalls häufige Schweißdrüsenkarzinome. Sie treten bei Patienten mittleren Alters auf. Es gibt keine eindeutige Geschlechterpräferenz. Die Kopfhaut, der Nacken und das Gesicht sind die häufigsten Lokalisationen, gefolgt von den Extremitäten.^33^ Die klinische Verdachtsdiagnose ist in der Regel eine Zyste. Die Tumoren können auf dem Boden eines vorbestehenden Hidradenoms oder *de novo* entstehen. In der Literatur wurde eine Metastasierung meist innerhalb der ersten zwei Jahre bei 36 %–60 % der Patienten beschrieben.^34^, ^35^ Im Fall einer Metastasierung steht die lymphatische Ausbreitung im Vordergrund. Als Risikofaktoren gelten vor allem die Tumorgröße, daneben eine schlechte Differenzierung und perineurale Invasion. Auch eine Arbeit aus 2015 hat bei zehgn Patienten mit Hidradenokarzinom innerhalb einer postoperativen Nachbeobachtungszeit von im Median 7 Jahren keine Metastasierung beobachten können.^33^ Einer weiteren, fast 300 Hidradenokarzinome umfassenden Untersuchung von Gao et al. zufolge, ist die Prognose des Hidradenokarzinoms deutlich besser als bisher angenommen.^36^ Fernmetastasen lagen in lediglich 2,4 % der Fälle vor. Das krebsspezifische 10‐Jahres‐Überleben betrug 90,5 %. Einfluss auf das krebsspezifische Überleben hatten einzig die Tumorgröße, nicht jedoch Lokalisation, Geschlecht, Alter, Ethnie, histologischer Differenzierungsgrad oder Art der chirurgischen Versorgung.^36^

Histologisch findet sich ein nodulärer, dermal‐subkutan gelegener Tumor, der abschnittsweise ein vorbestehendes Hidradenom erkennen lässt. Man beobachtet Zellatypien, ein infiltrierendes Tumorwachstum und Tumornekrosen. Insbesondere eine hohe Zahl an Mitosen (Ki‐67 > 11 %) gilt als Hinweis auf ein Hidradenokarzinom.^36^ Klarzellige und muzinöse Abschnitte sowie squamöse Metaplasien kommen vor. Da die karzinomatöse Entwicklung mitunter fokal in vorbestehenden Hidradenomen auftritt, empfiehlt sich bei Hidradenomen eine vollständige Aufarbeitung der Tumoren. Dennoch sind sehr selten auch eindeutig benigne Hidradenome beschrieben, die ausschließlich in die Lymphknoten metastasierten. Es handelt sich wohl ähnlich wie bei einigen Spitztumoren oder den metastasierenden Leiomyomen des Uterus um ein Spektrum von Tumoren, die Lymphknotenmetastasen aber keine weitere Ausbreitung aufweisen.^37^ Bei fünf Patienten mit klarzelligen Hidradenomen kam es nach chirurgischer Entfernung in keinem Fall zu einer Metastasierung innerhalb einer Nachbeobachtungszeit von 2 bis 11 Jahren. Klarzellige Hidradenome mit intralymphatischen Tumorzellen sollten als „atypische klarzellige Hidradenome“ bezeichnet und betroffene Patienten enger kontrolliert werden. Zudem sollten atypische Hidradenome wegen möglicher histopathologischer Abgrenzungsschwierigkeiten zu einem frühen Hidradenokarzinom vollständig exzidiert werden. Die Abgrenzung zum Hidradenom ist darüber hinaus auch bei gut differenzierten Hidradenokarzinomen erschwert.^38^ Insbesondere eine oberflächliche oder partielle Entfernung kann zu Fehldiagnosen führen.^38^ Bei axillär gelegenen Tumoren mit der Histomorphologie eines Hidradenokarzinoms muss differenzialdiagnostisch eine Metastase oder ein Ausläufer eines primären Mammakarzinoms abgegrenzt werden.^39^, ^40^

#### Extramammäres Paget‐Karzinom

Das extramammäre Paget‐Karzinom (auch extramammärer Morbus Paget) ist ein seltenes apokrin differenziertes intraepitheliales Schweißdrüsenkarzinom und tritt bevorzugt inguinal, perianal, perigenital und axillär auf. Insbesondere genitale Lokalisationen führen nicht selten klinisch zur initialen Fehldiagnose als entzündliche Dermatose.

Histopathologische Befunde eines Paget‐Karzinoms in der Anogenitalregion müssen differenzialdiagnostisch an eine Ausbreitung kolorektaler und urogenitaler Karzinome *per continuitatem* denken lassen und entsprechende Durchuntersuchungen nach sich ziehen. Einer aktuellen auf retrospektiven Daten basierenden Leitlinie US‐amerikanischer Kollegen zufolge, sollte das Screening nach einem anderweitigen Adenokarzinom entsprechend des anatomischen Subtyps erfolgen.^41^ So waren das perianale Paget‐Karzinom mit einer hohen Rate an Adenokarzinomen (25 %, hauptsächlich kolorektal) assoziiert, hingegen penoskrotale und vulväre Paget‐Karzinome nur in 6 % (hauptsächlich urogenital). Vorgeschlagen wird ein Screening‐Algorithmus, der beim perianalen Paget‐Karzinom eine Koloskopie, Urinzytologie und Computertomographie von Thorax, Abdomen und Becken umfasst sowie beim penoskrotalen Paget‐Karzinom eine Urinzytologie, einen Hämoccult‐Test und einen Prostata‐spezifischen Antigentest (insbesondere bei Personen unter 70 Jahren). Beim vulvären Paget‐Karzinom werden eine Urinzytologie und Mammographie empfohlen.

Histologisch zeigen sich intraepithelial einzelne und zum Teil in kleinen Gruppen (nestförmig) gelegene, größere, helle Zellen (Paget‐Zellen), mit PAS‐positivem und teils Muzin enthaltendem Zytoplasma, die sich invasiv ausbreiten können. Duktale Strukturen innerhalb der Nester und eine Positionierung unter Erhalt der keratinozytären Basalzellreihe sind recht typisch. Die seitliche Begrenzung ist beim Paget‐Karzinom sehr unscharf (*skipped areas*), zudem kann eine Ausbreitung entlang der Adnexstrukturen stattfinden, was die Beurteilung der Vollständigkeit der Exzision erschwert und die hohen Rezidivraten erklärt.^42^ Immunhistochemisch markieren sich die Zellen eines extramammären Paget‐Karzinoms mit CK 7 und CAM 5.2, was meist auch die Abgrenzung zum pagetoiden Morbus Bowen erlaubt und ggf. auch für die Schnittrandkontrolle genutzt werden sollte. Da auch Melanome eine pagetoide Ausbreitung in der Epidermis aufweisen können und die Paget‐Zellen zum Teil auch nestförmige Strukturen imitieren, empfiehlt sich zur Abgrenzung die immunhistochemische Färbung mit melanozytären Markern. Immunhistochemische Marker wie CK 20 und CDX2 können zudem Hinweise für ein zugrunde liegendes anderweitiges Karzinom sein.^43^, ^44^, ^45^, ^46^

#### Mikrozystisches Adnexkarzinom

Das mikrozystische Adnexkarzinom ist ein langsam wachsendes, lokal stark infiltrierendes, niedrigmalignes Schweißdrüsenkarzinom, möglicherweise auch ein Tumor mit zugleich follikulärer und Schweißdrüsendifferenzierung. Die Inzidenz wird mit 1,6 bis 6,5 Fällen pro 10 000 000 Menschen pro Jahr angegeben. Die Tumoren entstehen zumeist zentrofazial bei älteren Patienten, Frauen überwiegen etwas.^47^ Ein Auftreten ist jedoch bereits ab dem 3. Lebensjahrzehnt beschrieben.^48^, ^49^ Prädilektionsstellen umfassen hauptsächlich die Kopf‐Hals‐Region, bevorzugt das Gesicht und hier in ersten Linie die Wangen. Gelegentlich findet man mikrozystische Adnexkarzinome am Rumpf, sehr selten an den Extremitäten. Die palpatorisch indurierten Plaques können bis mehrere Zentimeter durchmessen, die Tumorgröße wird klinisch aufgrund des infiltrativen Wachstums in der Regel unterschätzt. Klinisch imponieren die Tumoren wie ein infiltrierend wachsendes Basalzellkarzinom. Die tief in die Subkutis infiltrierenden Karzinome zeigen häufig perineurale Tumorausläufer, die eine Ursache für die hohe lokale Rezidivrate bei inadäquater Exzision darstellen. Eine mikrographisch kontrollierte Exzision oder Exzision mit Sicherheitsabstand ist erforderlich. Eine Metastasierung wird nur außerordentlich selten bei immunsupprimierten Patienten und dann in die lokoregionalen Lymphknoten beobachtet.^50^, ^51^

Histopathologisch zeichnet sich das mikrozystische Adnexkarzinom durch dermal bis subkutan gelegene Tumorzellverbände ohne Verbindung zur Epidermis aus. In den oberen Abschnitten des Tumors sieht man größere zystisch dilatierte Schweißdrüsenausführungsgänge, die an kleine infundibuläre Zysten (Milien) erinnern. In den tiefen Abschnitten hingegen, zum Teil bis in die Subkutis reichend, imponieren kleine syringoide Epithelverbände ohne zytomorphologische Auffälligkeiten oder Mitosen sowie auch elongierte tubuläre Strukturen in einem hyalinen, sklerosierten oder auch muzinösen Bindegewebe. Typisch ist eine perineurale Ausbreitung in der Tumorperipherie. Fleckförmig findet man lymphozytäre Infiltrate. Etablierte immunhistochemische Marker zur Abgrenzung der histologischen Differenzialdiagnosen stehen nicht zur Verfügung. Das mikrozystische Adnexkarzinom ist Ber‐EP4‐negativ oder nur fokal membranös angefärbt. Insgesamt stellen die Infiltration der Subkutis und die perineurale Ausbreitung die wichtigsten diagnostischen Hinweise zur Abgrenzung vor allem vom Syringom und desmoplastischen Trichoepitheliom dar. CK 20‐positive Merkelzellen finden sich nicht, was gelegentlich bei der Unterscheidung vom desmoplastischen Trichoepitheliom helfen kann. Ein wichtiger Aspekt ist die Abgrenzung zu syringoiden hamartomatösen Proliferationen bei einigen familiär gebundenen Syndromen wie zum Beispiel dem Rombo‐Syndrom und Nikolau‐Balus‐Syndrom. Bei diesen zumeist auch jüngeren Patienten finden sich klinisch an ein mikrozystisches Adnexkarzinom erinnernde Plaques auf beiden Wangen. Diese sind im Gesicht zumeist begleitet von Teleangiektasien, milienförmigen Zysten und/oder einer Atrophodermia vermiculata, Veränderungen, die sich ebenfalls auf den Oberarmen der Patienten finden können. Die für das mikrozystische Adnexkarzinom typische perineurale Infiltration ist jedoch nicht nachweisbar. Die korrekte Diagnose an einer repräsentativen Gewebeentnahme ist essentiell, um nachfolgende ausgedehnte mutilierende operative Eingriffe zu vermeiden.^52^, ^53^ Vier Gene aus dem Calciumsignalweg sind auf RNA‐ und Proteinebene beim mikrozystischen Adnexkarzinom hochreguliert: *CACNA1S*, *ATP2A1*, *RYR1* und *MYLK3* und könnten zukünftig als diagnostische Marker dienen.

#### Digitales papilläres Adenokarzinom

Das digitale papilläre Adenokarzinom ist eine Erkrankung des Erwachsenenalters mit einem Erkrankungsgipfel in der 5. Lebensdekade; Männer sind häufiger betroffen als Frauen.^54^, ^55^ In Einzelfällen erkranken jedoch auch deutlich jüngere Patienten. Klinisch finden sich langsam wachsende, solitäre Knoten an Fingern oder Zehen, zum Teil an Fußsohlen und Handflächen. Ursprünglich wurde ein digitales papilläres Adenom vom digitalen papillären Adenokarzinom abgegrenzt, es zeigte sich jedoch im Verlauf, dass eine sichere Prognose anhand von histologischen Merkmalen und Differenzierungsgrad nicht möglich ist.^54^, ^56^ Kürzlich wurde in einer größeren Studie in 96 % der untersuchten digitalen papillären Adenokarzinome humanes Papillomavirus 42 (HPV42) nachgewiesen, ein zuvor als *low‐risk* eingeordneter HPV‐Typ.^8^, ^57^ Die HPV42 induzierte Transformation ruft ein keimzellähnliches Transkriptionsprogramm hervor, welches in allen HPV‐positiven Karzinomen konserviert ist und gewissermaßen einen „Fingerabdruck“ onkogener HPV darstellt. Durch den Nachweis von HPV42 wurden digitale papilläre Adenokarzinome zunehmend auch in extraakraler Lokalisation nachgewiesen und das morphologische Spektrum dieser Neoplasien erweitert.

Das digitale papilläre Adenokarzinom neigt zu Lokalrezidiven. Gemäß Literaturdaten treten in 14 % bis 41 % der Patienten, vielfach erst nach Jahren, Metastasen auf, insbesondere in Lymphknoten und Lunge.

Histologisch zeigt sich ein dermal bis subkutan gelegener Tumor ohne Epidermisbezug mit zystischem oder solid‐zystischem Aufbau.^56^ Die Mehrheit der Tumoren erscheint mit gut umschriebenen nodulären Konturen auf den ersten Blick blande and harmlos. Die überwiegend basophilen Tumorzellen zeigen moderate zytologische Atypien, bilden vielfach papilläre Strukturen und eine Rücken‐an‐Rücken Lagerung neoplastischer Formationen. Die papillären Strukturen können aber auch völlig fehlen. In der Regel finden sich einige Mitosen. Squamöse Metaplasien und eine Klarzelldifferenzierung können auftreten. Letztlich ist das morphologische Spektrum breit und reicht von einem Hidradenom‐ oder Zystadenom‐ähnlichen Bild bis hin zu hochpleomorphen infiltrativen Epithelproliferaten. Die Tumoren können ganz solide oder extrem zystisch sein und damit an Hidrozystome erinnern, die akral quasi nicht auftreten.

Der HPV42‐Nachweis ist sehr hilfreich in der differenzialdiagnostischen Abgrenzung. Immunhistochemisch färben sich die zentral gelegenen drüsigen Zellverbände inhomogen CK 7 positiv. Eine erhöhte Proliferation (Ki‐67) ist nachweisbar. Typisch ist eine Kompartimentierung mit dem Nachweis einer zweiten Population myoepithelialer Zellen auch in differenzialdiagnostischer Abgrenzung von zum Beispiel nodulären Hidradenomen.

#### Adenoid‐zystisches Karzinom

Das adenoid‐zystische Karzinom ist ein seltener Hautadnextumor, der etwas häufiger bei Frauen diagnostiziert wird, üblicherweise in der 6. Lebensdekade.^58^, ^59^ Es ist meist in der Kopf‐Hals‐Region lokalisiert, häufig im äußeren Gehörgang, gelegentlich am Rumpf oder genital.^60^ Lokalrezidive können auftreten, Lymphknotenmetastasen sowie auch Fernmetastasen (vor allem Lunge) sind äußerst selten, können jedoch auch erst nach vielen Jahren in Erscheinung treten.^61^, ^62^, ^63^ Bei Tumoren im äußeren Gehörgang ist das Metastasierungsrisiko allerdings mit etwa 30 % erhöht und dem der gleichnamigen Speicheldrüsentumoren vergleichbar. Auch genital wurde eine höhere Rezidiv‐ und Metastasierungsrate berichtet. Das 5‐Jahres‐ und 10‐Jahres‐Überleben werden in einer Studie an 201 Patienten mit 87,0 % beziehungsweise 76,0 %, angegeben.^59^ 65,8 % der Patienten hatten einen lokalisierten Befund (Stadium I und II).

Histologisch ist das kutane adenoid‐zystische Karzinom analogen Tumoren der Speicheldrüsen identisch. Es finden sich duktale und modifizierte myoepitheliale Zellen, die tubuläre, kribriforme und solide Wachstumsmuster ausbilden. Typisch sind aggregierte Tumorknoten mit multiplen Muzin‐gefüllten Zysten (adenoid‐zystisches Muster) und eine relativ blande Zytologie. Die Tumoren können infiltrativ in das umgebende Gewebe wachsen. Bei perineuraler Ausbreitung zeigt sich eine erhöhte Rezidivrate (76 %).

Immunhistochemisch findet sich neben der Expression von CK 7 typischerweise auch eine Expression von myoepithelialen Markern. Molekulargenetisch wurde eine *MYBL1*‐Fusion nachgewiesen.^64^, ^65^ Eine 1p36‐Deletion, die bei adenoid‐zystischen Karzinomen der Speicheldrüsen als prognostisch ungünstig gilt, konnte bei kutanen adenoid‐zystischen Karzinomen bisher nicht gefunden werden.^66^

Differenzialdiagnostisch ist je nach Lokalisation eine kutane Ausbreitung eines extrakutan glandulären Tumors auszuschließen. Extrakutane adenoid‐zystische Karzinome treten neben den Speicheldrüsen auch in Tränendrüsen, Nasennebenhöhlen, Kehlkopf, Bronchien, Lunge, Mamma, Cervix, Bartholin‐Drüsen und Prostata auf. Eine kutane Metastasierung eines extrakutanen adenoid‐zystischen Karzinoms stellt allerdings eine absolute Seltenheit dar.^67^

**TABELLE 1:** Klinische, histopathologische und molekularpathologische Charakteristika sowie differenzialdiagnostische Besonderheiten von Schweißdrüsenkarzinomen.

	Klinik	Alter	Lokalisation	Histologische Charakteristika	Immunhistochemische Besonderheiten	Molekularpathologie Mutationen und Copy‐Number‐Variationen	Differenzialdiagnostische Aspekte
*Schweißdrüsenkarzinom Subtypen mit benignen Pendants*							
***Porokarzinom*** (*Syn*.: malignes Porom, epidermotropes ekkrines Karzinom, malignes Hidroacanthoma simplex)	Erinnern an Plattenepithel‐ oder Basalzellkarzinome	Überwiegend ältere Patienten	44 % untere Extremitäten, 24 % Stamm, 24 % Kopf‐Hals	Atypische poroide Zellen, fokal duktale Differenzierung, zahlreiche Mitosen, großflächige Tumornekrosen möglich; klarzellige und squamoide Differenzierungen nicht selten Pigmentierung durch Kolonisierung mit Melanozyten intralymphatische Ausbreitung in der Tumorperipherie	*Positiv*: CK7, NUT	Ca. 280 berichtete Tumoren: Generell high TMB, meist auf Signaturen von Mutationsprozessen im Zusammenhang mit UV‐Strahlung zurückzuführen *YAP1*‐Fusionsgene *PAK1*‐Fusionsgene *PAK2*‐Fusionsgene *PAK3*‐Fusionsgene Andere Fusionsgene: 1 x *WWTR1::NUTM1*, 1 x *PIK3R1::YTHDC2* 8 x 1.6 Mb Amplification in 11q13.3 (beinhaltet die Gene *CCND1*, *FGF3*, *FGF4*, *FGF19*) 7 x 6.9 Mb Deletion at 9p21.3 (beinhaltet die Gene *CDKN2A/B*, *MTAP*, *TEK*, *MOB3B*) 44 x *TP53*‐Mutationen 14 x *HRAS*‐Mutationen 11 x *KMT2D*‐Mutationen 11 x *LRP1B*‐Mutationen 9 x *CDKN2A*‐Mutationen 9 x *PIK3CA*‐Mutationen	*Necrosis en mass*‘ auch bei Poromen möglich malignes Hidroacanthoma simple X muss histologisch von einem pagetoiden Morbus Bowen und der klonalen seborrhoischen Keratose abgegrenzt werden
***Hidradeno‐*** ***karzinom*** (*Syn*.: klarzelliges ekkrines Hidradenokarzinom, malignes klarzelliges Akrospirom, malignes ekkrines Akrospirom)	Asymptomatische indurierte, erythematöse Knoten	Mittleres Alter	40 % okzipital 29 % Gesicht 30 % Extremitäten	Dermal‐subkutaner nodulärer Tumor zytologische Atypien, infiltrierendes Tumorwachstum und Tumornekrosen hohe Zahl an Mitosen klarzellige, muzinöse Abschnitte und squamöse Metaplasien möglich	*Positiv*: CK7, MAML2 Fusion	130 berichtete Tumoren: 12 x *CRTC1::MAML2*‐Rearrangement 9 x *MAML2‐*Rearrangement 9 x MSS 6 x high TMB 5 x low TMB 2 x mögliche unbalancierte Translokation / Disruption von *CRTC1* 9 x *EGFR* Disomie, 3 x Polysomie, 6 x trisomie 2 x *Her2/neu* Amplifikation	Karzinomentwicklung mitunter fokal in vorbestehenden Hidradenomen, vollständige Aufarbeitung empfohlen atypische Hidradenome (insb. “atypische klarzellige Hidradenome” mit intralymphatischen Tumorzellen) sollten wegen histopathologischer Abgrenzungsschwierigkeiten zu einem frühen Hidradenokarzinom in toto exzidiert werden bei axillären Tumoren mit der Histomorphologie eines Hidradenokarzinoms an Metastase/Ausläufer eines primären Mammakarzinoms denken
***Maligner Mischtumor der Haut*** (*Syn*.: maligner apokriner Mischtumor der Haut, malignes chondroides Syringom der Haut) ***Myoepitheliales Karzinom der Haut*** (*Syn*.: malignes kutanes Myoepitheliom)^68^	subkutane Knoten mit möglicher Ulzeration und Verwachsung mit dem Untergrund entwickeln sich aus vorbestehenden Mischtumoren bzw. Myoepitheliomen der Haut	Breites Altersspektrum, medianes Alter 50 J.	Meist Extremitäten (Hände, Füße), auch Gesicht (myoepitheliales Karzinom)	In Teilen noch einem vorbestehenden Mischtumor entsprechend, jedoch überwiegend deutlich infiltrierend wachsend, noch erkennbare glanduläre und tubuläre Strukturen, elongierte, verzweigte Tubuli und Dekapitationssekretion möglich im Stroma abschnittsweise noch hyaline, kartilaginäre und ossäre Strukturen, überwiegend myoepitheliale Differenzierung beim myoepithelialen Karzinom (duktale Strukturen per definitionem abwesend)	*Positiv*: PLAG1, selterner HMGA2 in benignen, apokrin differenzierten Anteilen^69^, ^70^, nukleär, variable Intenstät^71^ EMA, Calponin, oft auch S100, CK, aSMA, teilweise GFAP (^72^	Keine bzw. keine rekurrenten genetischen Veränderungen berichtet	Kontinuum vom malignen Mischtumor zum myoeithelialen Karzinom analog dem Spektrum der benignen Pendants bei Nähe zu Speicheldrüsen wichtige Differenzialdiagnose maligner Mischtumoren der Speicheldrüsen (Karzinom aus pleomorphem Adenom)
***Spiradenokarzinom /*** ***Zylindrokarzinom*** ***Spiradenozylindrokarzinom*** ^73^ (alle drei stellen die morphologischen Enden eines gemeinsamen Tumorspektrums dar)	schnell wachsend, ulzerierende Knoten, in vorbestehenden Spiradenomen / Auf dem Boden multipler Zylindrome bei Brooke‐Spiegler‐Syndrom	In der Regel > 50 Lebensjahr; medianes Alter 63 J.; im jüngeren Erwachsenenalter möglich	Häufig Extremitäten, auch Kopf‐Hals‐Lokalisation / hauptsächlich Kopf und Hals	Nur zu diagnostizieren, wenn in Teilen noch ein Spiradenom / Zylindrom erkennbar ist häufig im tiefen Korium und oberer Subkutis, infiltrierendes Wachstum in Subkutis möglich Verlust der PAS‐positiven Basalmembran beim Zylindrokarzinom erhöhte Zelldichte, deutliche zytologische Atypien, zahlreiche Mitosen, teilweise Spindelzellen häufig Nekroseareale	Verlust der PAS+ hyalinen Membranen positiv: CK7, durchgängig p53 in den malignen Abschnitten	63 berichtete Spiradenokarzinome: 6 x *TP53*‐Mutationen 5 x *TERT*‐Mutationen (hotspot *C228T*, *C250T*) Signaturen assoziiert Mit DNA MMR high‐grade Spiradenokarzinome weisen große CNVs auf (für Zylindrokarzinom und Spiradenozylindrokarzinom keine od. keine rekurrenten genetischen Veränderungen berichtet)	Einblutungen und begrenzte zytologische Atypien und Mitosen in degenerativ veränderten Spiradenomen, jedoch keine ausgedehnteren Tumornekrosen
***Syringocystadenocarcinoma*** ***papilliferum*** ^74^	Erythemtöse Knoten oder Plaque hauptsächlich Entwicklung aus einem Syringocystadenoma papilliferum	Mehrheitlich ältere Patienten, durchschnittlich 6. Lebensdekade	Häufig Kopf‐Hals‐Lokalisation	Syringocystadenoma papilliferum noch erkennbar infiltrierendes Wachstum tubulärer Strukturen mit zellulären Atypien Tumornekrose möglich		15 berichtete Tumoren: 5 x *HRAS*‐Mutationen (davon 4 x G13R) 5x *TP53*‐Mutationen	

Schweißdrüsenkarzinom Subtypen ohne benigne Pendants							
***Extramammäres*** ***Paget‐Karzinom*** (*Syn*.: extramammärer Morbus Paget)	Unscharf begrenzte, z.T. erosive, erythematöse Plaque klinisch an eine entzündliche Dermatose erinnernd klinsch inapperente Ausläufer	Durchschnittliches Manifestationsalter 70 J.	Vor allem inguinal, perianal, perigenital und axillär	Intraepithelial einzelne und in kleinen Gruppen gelegene, größere, helle Zellen mit PAS‐positivem Zytoplasma, was Muzin enthalten kann duktale Strukturen innerhalb der Nester Positionierung der Nester oberhalb einer erhaltenen keratinozytären Basalzellreihe unscharfe seitliche Begrenzung Ausbreitung entlang der Adnexstrukturen und die tiefer gelegende Dermis	*Positiv*: homogen CK 7 und CAM 5.2	> 500 berichtete Tumoren: bis zu 43 % Amplifikationen und 15 % rekurrente Punktmutationen in *ERBB2* konstitutive Aktivierung des PI3K‐AKT‐mTOR‐Stoffwechsels, häufig bedingt durch Mutationen in *PI3KCA* (35 %, *p.E542K*‐Variante) und *AKT1* mehrere Studien: *TOP2A*‐Amplifikation in bis zu 40 % der Fälle mehrere Studien: Mutationen in p53 in etw 30 % der Fälle ‐ verbunden mit fortgeschrittenem Stadium und schlechterer Prognose mehrere Studien: homozygote Deletionen von CDKN2A in 25 % der Fälle, assoziiert mit höheren Rezidivraten niedrige TMB, selten MSI (< 1 %)	Mögliche Ausbreitung per continuitatem kolorektaler und urogenitaler Karzinome in der Anogenitalregion; Screening nach einem zugrundliegenden Adenokarzinom vor allem beim perianalen Paget‐Karzinom (deutlich seltener bei periskrotalem und perivulvärem) CK 7 und CAM 5.2 Positivität erlauben die Abgrenzung zum pagetoiden Morbus Bowen und in situ Melanom (ggf. auch Einsatz melanozytärer Marker) CK 20 und CDX2 hinweisend für ein zugrunde liegendes anderweitiges Karzinom
***Mikrozystisches*** ***Adnexkarzinom*** (*Syn*.: sklerosierendes Schweißdrüsenkarzinom, ekkrines Epitheliom, syringomatöses Karzinom)	Langsam wachsende, indurierte, Plaque bis zu mehrere Zentimeter durchmessend	Ab dem 3. Lebensjahrzehnt; häufiger ältere Patienten	Kopf‐Hals (bevorzugt Wangen), selten Rumpf oder Extremitäten	Dermal bis subkutan gelegene Tumorzellverbände ohne Verbindung zur Epidermis in oberen Abschnitten größere zystisch dilatierte Schweißdrüsenausführungsgänge wie Milien imponierend; in den tiefen Abschnitten kleine syringoide Epithelverbände oder elongierte tubuläre Strukturen ohne zytomorphologische Auffälligkeiten oder Mitosen in einem hyalinen, sklerosierten oder auch muzinösen Stroma perineurale Ausbreitung in der Tumorperipherie	*Positiv*: CK7 negativ: Ber‐EP4 (oder nur fokal membranös positiv)	25 berichtete Tumoren: 5x *JAK/STAT* signaling pathway, calcium signaling pathway, *cGMP‐PKG* signaling pathway enriched 7x CNVs in *JAK2*, *MAF*, *MAFB*, *JUN*, *FGFR1*	Abgrenzung v.a. zu infiltrierend wachsenden Basalzellkarzinomen Infiltration der Subkutis und perineurale Ausbreitung als wichtigste Hinweise zur Abgrenzung v.a. vom Syringom und desmoplastischen Trichoepitheliom Fehlen CK20‐positiver Merkelzellen zur Unterscheidung vom desmoplastischen Trichoepitheliom Abgrenzung zu syringoiden hamartomatösen Proliferationen bei familiär gebundenen Syndromen typischerweise bei jüngeren Patienten
***Digitales papilläres Adenokarzinom***	Langsam wachsender, solitärer Knoten	Erwachsen, Gipfel in der 5. Lebensdekade; Männer häufiger	Finger, Zehen, selten plantar / palmar	Dermaler bis subkutaner, gut umschriebener Tumor ohne Epidermisbezug zystischer oder solid‐zystischer Aufbau überwiegend basophile Tumorzellen mit moderaten zytologischen Atypien; bilden vielfach papilläre Strukturen; papilläre Strukturen können fehlen; einige Mitosen squamöse Metaplasie und Klarzelldifferenzierung möglich letztlich breites morphologisches Spektrum von einem Hidradenom‐ oder Zystadenom‐ähnlichem Bild bis hin zu hochpleomorphen infiltrativen Epithelproliferaten	Kompartimentierung von CK7‐positiven epithelialen und Aktin‐positiven myoepithelialen Zellen	Ca. 136 berichtete Tumoren: nahezu alle HPV42 positiv 10 x *TP53*‐Mutationen 9 x *BRAF*‐Mutationen 7 x *EGFR*‐Überexpression 6 x Mutationen in *KMT2B*, *MAML2*, *MAP3K1*, *CACNA1E*, *PDGFRA*, *HIF1A*, *PRDM16*, *TCF7L1* 4 x Anreicherung von Genen in Verbindung mit Matrix Remodelierung, Metabolismus, DNA Reparatur 2 x *CRTC3::MAM2* Rarrengements 2 x *TRPS1::PLAG1* Rearrengements	Fehlende Kompartimentierung z. B. im Hidradenom
***Adenoid‐zystisches Karzinom***	Derber, rötlicher oder hautfarbener Knotendermal und/oder subkutan	Üblicherweise in der 6. Lebensdekade; etwas häufiger Frauen	Meist in Kopf‐Hals‐Lokalisation, häufig im äußeren Gehörgang, gelegentlich am Rumpf oder genital	Analogen Tumoren der Speicheldrüsen identisch duktale und modifizierte myoepitheliale Zellen, die tubuläre, kribriforme und solide Wachstumsmuster ausbilden, wachsen infiltrativ in das umgebende Gewebe typisch aggregierte Tumorknoten mit multiplen Muzin‐gefüllten Zysten relativ blande Zytologie	*Positiv*: CK 7, myoepitheliale Marker SOX10 intensiv, diffus) und MYB (70–100 %) CD117 diffus (> 50 %) aSMA, CK15 (36 %), Vimentin (57 %)^75^	77 berichtete Tumoren: 17 x *MYB* Expression 16x *MYB* Rearrangement 6 x *MYB‐NFIB* Fusion 3x *MYBL1* Expression 2 x *MYBL1‐NFIB* Fusion	Ausbreitung extrakutaner adenoid‐zystischer Karzinome der Speicheldrüsen, Tränendrüsen, Nasennebenhöhlen, Kehlkopf, Bronchien, Lunge, Mamma, Cervix, Bartholin‐Drüsen und Prostata (Topographie!) sehr selten sekundär kutane Metastasierung eines extrakutanen adenoid‐zystischen Karzinoms
***Muzinöses Karzinom der Haut*** ^76^, ^77^, ^78^ ***Endokrines muzinproduzierendes Schweißdrüsenkarzinom*** ^79^, ^80^ (a.e. dem Formenkreis des kutanen muzinösen Karzinoms zuzuordnen)	Solitäre, erythematöse Knoten Kleine, derbe, rötliche Knoten	Erwachsene; etwas häufiger Frauen	Überwiegend Kopf‐Hals‐Region, insbesondersperiorbital Periorbitalregion, besonders Augenlider	Dermal gelegener, unscharf begrenzter Tumor aus mehreren Knoten; Nester, solide Formationen und kribriforme Tumorzellverbände in reichlich muzinösem Stroma; teilweise duktale Differenzierung und in‐situ‐Komponente Umschrieben, multinodulär; solide, papilläre zystische Verbände; uniforme basaloide Tumorzellen; kleine Muzinseen	*Positiv*: CK7, EMA, CEA, Hormonrezeptoren; teilweise myoepitheliale Lage (In‐situ‐Komponente) negativ: CK20 *Positiv*: BerEP4, CK7, CAM5.2, Synaptophysin, Chromogranin, Hormonrezeptoren, MYB, INSM1	Keine bzw. keine rekurrenten genetischen Veränderungen berichtet lediglich diploide DNA mit minimalen CNAs, niedrige TMB, selten MSI, keine MMR‐Defizienz Keine bzw. keine rekurrenten genetischen Veränderungen berichtet	Tumoren am Rumpf und axillär klinisch ggf. hinweisend auf ein metastasierendes muzinöses Karzinom der Mamma in Genital‐ und Anallokalisation entsprechend metastasierende muzinöse Kolorektalkarzinome (CK20 oder CDX2 positiv) Abgrenzung von Basalzellkarzinomen zum muzinösen Karzinom ist schwierig, hinweisend sind Verbindung mit Epidermis, Spaltbildungen zum Stroma und Palisadierungen der Zellkerne; Hybridformen sind möglich
***Kutanes adenosquamöses Karzinom*** ^81^, ^82^, ^83^, ^84^ (*Syn*.: squamoides ekkrines duktales Karzinom)	Häufig ulzerierter Knoten oder Plaque in aktinisch geschädigter Haut	Insbesondere bei älteren Männern	In aktinisch geschädigter Haut	Biphasische Tumoren mit oberflächlich squamöser Plattenepithelkarzinom‐ und tiefer duktaler Karzinomkomponente mit desmoplastischem Stroma infiltrierend wachsend deutliche Atypien, zahlreiche Mitosen perineurale und lymphovaskuläre Infiltration	*Positiv*: Darstellung duktaler Differenzierung mit EMA und CEA	16 berichtete Tumoren: generell high TMB UV‐assoziierte Mutationen 10 x *TP53*‐Mutationen 8 x *NOTCH1*‐ und *NOTCH2*‐Mutationen überexprimierte Gene: assoziiert mit Schweißdrüsendifferenzierung (*KRT31*, *KRT77*, *DCD*, *PIP*, *SCGB2A2*) & Speicheldrüsendifferenzierung (*AMY1C*, *DNASE2B*)	Keine duktale Komponente im Plattenepithelkarzinom; keine deutlichen Atypien im mikrozystischen Adnexkarzinom; poroide Zytologie und fehlende biphasische Komponente im Porokarzinom
***Kutanes (apokrines) kribriformes Karzinom*** ^85^, ^86^, ^87^, ^88^ (*Syn*.: kribriformer Tumor)	Solitäre, klinisch gutartig erscheinende, derbe Knötchen oder Knoten bis 3cm	Medianes Patientenalter 47 J.; etwas häufiger bei Frauen	Häufig Extremitäten	Tief dermal und subkutan, kein Epidermisbezug helle, eosinophile Tumorzellen, geringe Atypien, wenige Mitosen peripher infiltrierendes Wachstum kribriformes Bild durch multiple Vakuolen in einzelnen Epithelverbänden mit dünnen fadenförmigen Septierungen in duktalen Lumina ohne Muzin	*Positiv*: Her2 (variabel), ER (variabel) negativ: Gata3, PR; keine myoepitheliale Komponente^85^, ^86^, ^87^	Keine bzw. keine rekurrenten genetischen Veränderungen berichtet	Können histomorphologisch als Mammakarzinom‐ oder Adenokarzinom‐Metastasen fehlinterpretiert werden; weitere DD: kutanes sekretorisches Karzinom
*Sekretorisches Karzinom der Haut* ^89^, ^90^, ^91^, ^92^	Dermaler/ subkutaner Knoten	3.–8. Lebensdekade^92^ etwas häufiger bei Frauen	Häufig axillär, auch Kopf‐Hals, Extremitäten, Rumpf	Gut umschrieben, dermal und subkutan, kein Epidermisbezug blande ovale, runde bis kuboidale Zellen mikrozystische und tubuläre Strukturen mit reichlich eosinophilem, PAS‐positivem Sekret, außerdem solide Areale fokal pseudopapilläre Strukturen, selten auch muzinhaltige Areale	*Positiv*: S100, Mammaglobin, STAT5, GATA3, NTRK	15 berichtete Tumoren: 7 x *ETV6* Rearrangement 7 x *ETV6::NTRK3* Fusion	Identischer Tumor an der Brustdrüse und in der Speicheldrüse beschrieben
***Mikrosekretorisches Karzinom der Haut*** 91	Kleine, knotige Tumoren	Ältere Patienten		Mikrozystische Tubuli mit basophiler luminaler Sekretion	*Positiv*: S100, SOX10, p63; herdörmige myoepitheliale Differenzierung negativ: Mammaglobin	*MEF2C::SS18* Fusion	Identischer Tumor in der Speicheldrüse beschrieben
)Siegelringzellkarzinom der Haut^93^, ^94^	Diffuse Lidschwellung, teilweise mitErythem (entzündliche Erkrankungen – Blepharitis, Chalazion ‐ imitierend); axillär umschriebene Plaque oder Knoten	Medianes Patientenalter 66 J.; deutlich häufiger bei Männern	2/3 der Tumoren Augenlider, 1/3 axillär	Strang‐ oder rasenartige Anordnung atypischer Zellen mit exzentrischen Nuklei und entweder vakuolärem (Siegelringzell‐Variante) oder eosinophilem, granulärem Zytoplasma (histiozytäre‘ Variante)	*Positiv*: Ber‐EP4, Androgenrezeptor; Podoplanin und p63 beim primären Siegelringzellkarzinom des Augenlides (nicht jedoch bei Augenlid‐Metastasen von anderen Tumoren) eher negativ: ER, PR	Mutationen von *DH1*, *PIK3CA*, *NTRK3*, *CDKN1B* und *TP53* sind beschrieben	Wichtige Abgrenzung von Metastasen eines invasiv‐lobulären Mammakarzinoms (Metastasierung in Augenlider mehrfach beschrieben) oder eines gastrointestinalen Adenokarzinoms mit Siegelringzell‐Morphologie – Durchuntersuchung notwendig
***Ekkrines duktales Karzinom NOS (stellt Ausschlussdiagnose dar)*** ^83^	Tumorknoten und Plaques	Erwachsene		Infiltrierende dermale Tumoren pleomorphe, polygonale, epitheloide Tumorzellen in häufig desmoplastischem Stroma, duktale Differenzierung perineurale und lymphovaskuläre Infiltration, Tumornekrosen	*Positiv*: Darstellung duktaler Differenzierung mit EMA und CEA	Keine bzw. keine rekurrenten genetischen Veränderungen berichtet	Ähnelt metastatischen Absiedlungen insbesondere eines Mammakarzinoms; repräsentiert eine Ausschlussdiagnose
***Kutanes NUT‐Karzinom*** ^95^, ^96^, ^97^	Dermaler /subkutaner Knoten	Meist 7.–9. Lebensdekade^95^; Frauen deutlich häufiger betroffen	Einzelfälle in Kopfhaut, Gesicht, Extremitäten und akral	Teilweise gering differenziert mit großen vesikulären Kernen, prominenten Nukleoli, Mitosen und Nekrosen^96^ *BRD3*‐Rearrangement‐Tumoren zeigen Verbindung zur Epidermis, kleinknotigen Aufbau und duktale Strukturen oder intrazytoplasmatische Lumina *BRD4*‐Rearrangement‐Tumoren zeigen großknotigen Aufbau	*Positiv*: NUT, p63, EMA, TRPS1, c‐MYB, CD56, CK und INSM1 variabel exprimiert, diffus SOX10 bei 75 %	*8 berichtete Tumoren*: *4 x BRD3::NUTM1 Fusion* *3 x BRD4::NUTM1 Fusion* *1 x BRD3::NUTM2B Fusion*	NUT‐Karzinom identische Tumoren sinonasal, nasopharyngeal und thorakal beschrieben, dort prognostisch schlechter, jüngere Patienten

*Abk*.: aSMA, alpha‐Glattmuskelaktin; Ber‐EP4, epitheliales Zelladhäsionsmolekül; CAM5.2, monoklonaler Mausantikörper gegen Zytokeratin; CD117, Tyrosinkinase‐Rezeptor KIT; CEA, carcinoembryonales Antigen; CK, Zytokeratin; CNA, *copy number alterations*; CNV, *copy number variations*; DD, Differenzialdiagnose; EMA, epitheliales Membranantigen; ER, Östrogenrezeptor; GATA3, *GATA binding protein 3*; GCDFP‐15, *gross cystic disease fluid protein 15*; GFAP, *glial fibrillary acidic protein*; HMGA2, *high mobility group AT‐hook 2*; HR, *hazard ratio*; J., Jahre; MAML2, *mastermind‐like transcriptional coactivator 2*; MYB, *myeloblastosis*; MSI, Mikrosatelliteninstabilität; NTRK, *neurotrophic tyrosine kinase receptor*; NUT, *nuclear protein in testis*; PLAG1, *pleomorphic adenoma gene 1*; PR, Progesteronrezeptor; Syn., Synonym; STAT5, *signal transducer and activator of transcription 5*; TMB, *tumor mutational burden* (Tumormutationslast); TRPS1, *trichorhinophalangeal syndrome 1*

### Genetische Alterationen

*Kapitelautoren*: Silke Redler, Lara Racz, Friedegund Meier, Jürgen C. Becker, Almut Böer‐Auer

Schweißdrüsenkarzinome stellen auch genetisch eine heterogene Gruppe seltener Tumoren dar. Auch wenn ein monogener Erbgang mit Keimbahnmutationen in einzelnen Familien nicht vollständig auszuschließen ist (beispielsweise beim Brooke‐Spiegler‐Syndrom), ist davon auszugehen, dass es sich bei Schweißdrüsenkarzinomen überwiegend um sporadische Tumoren handelt, die nicht im Rahmen eines erblichen Tumorrisikosyndroms auftreten. Die Entstehung von sporadischen Tumoren geht in einem mehrstufigen Prozess mit dem kumulativen Erwerb somatischer Mutationen in verschiedenen Genen sowie anderweitigen komplexen genomischen Alterationen einher.

In der Tabelle 1 sind die wichtigsten genetischen Veränderungen der verschiedenen Schweißdrüsenkarzinomentitäten und bis dato identifizierte somatische therapeutische Zielstrukturen zusammengefasst. Es werden nur rekurrente genetische Befunde aufgeführt.

Während für andere maligne Hauttumoren umfangreiche molekulargenetische Erkenntnisse vorliegen, sind die molekulargenetischen Kenntnisse über Schweißdrüsentumoren bis dato gering. Lediglich für einzelne Entitäten ergibt sich ein umfassenderes Bild, das Einblick in die Pathogenese sowie Klassifizierung dieser Tumorentitäten ermöglicht und damit die Option gezielter Therapieansätze und individualisierter Präzisionsmedizin eröffnet. Beispielsweise konnten spezifische molekulare Veränderungen – insbesondere beim ekkrinen Porokarzinom – bereits für vielversprechende Therapieansätze genutzt werden.

Präzisionsmedizin gewinnt für die Tumortherapie zunehmend an Bedeutung, insbesondere für seltene Tumorentitäten wie Schweißdrüsenkarzinome, für die bislang keine evidenzbasierten, leitlinienkonformen Therapiestandards existieren.

Die angewandten und in der Tabelle 1 zusammengefassten molekularen Untersuchungsmethoden bei Schweißdrüsenkarzinomen sind inhomogen und reichen von der Bestimmung einzelner genetischer Varianten, der gezielten Analyse einzelner, klar umschriebener Regionen hin zu weit gefassten genetischen Analysen mit Hilfe sogenannter Genpanels oder Gesamtexomanalysen (whole exome sequencing, WES), sowie verschiedenartigen Genexpressionsanalysen.

Die Anzahl der in den jeweiligen Studien untersuchten Tumoren ist unterschiedlich, aber im Vergleich mit häufigen Tumorentitäten insgesamt gering. In einigen Studien wurde alleinig die Tumor‐DNA (Tumor‐only‐Untersuchungen) untersucht, in anderen Studien wurde eine parallele Analyse von Tumoren und konstitutionellen Varianten (in der Regel in Form von umgebender tumorfreier Haut) durchgeführt. Die untersuchten Kollektive sind zu klein, um inter‐ und intragenetische Heterogenität dieser Tumorentitäten abschließend zu bewerten.

Es ist ein zentrales Anliegen, die methodischen Entwicklungen in Form von perspektivisch Ganzgenomanalysen von Tumoren auch bei Schweißdrüsenkarzinomen anzuwenden. Diese Analysen sollten in einem qualitätsgesicherten Umfeld erfolgen und umfassen einen Prozess, der von der Präanalytik des Probenmaterials über die Sequenzierung bis zur Identifizierung, Annotation und Klassifikation sowie klinischen Interpretation von Varianten reicht. Relevant ist die Einbettung in einen klinischen Kontext, der unter anderem die Indikationsstellung, Beratungsleistungen und gegebenenfalls Beratungen durch das interdisziplinäre Organboard oder das molekulare Tumorboard umfasst. Dies schließt eine dezidierte Kenntnis der klinischen Studienlandschaft und der aktuellen Zulassungssituationen ein. Die Einbindung der Humangenetik ist empfehlenswert. Durch den zunehmenden Einsatz einer umfangreichen Analytik sowohl an Tumor‐DNA als auch an angrenzendem Normalgewebe, steigt die Wahrscheinlichkeit, Varianten zu detektieren, die möglicherweise erbliche (konstitutionelle) Varianten darstellen und eine Gendiagnostikgesetz‐konforme Beratung der Patienten und Familienangehörigen durch einen Facharzt für Humangenetik erforderlich machen.

### Dermatoskopie und andere Verfahren der In‐vivo‐Bildgebung

*Kapitelautor*: Michael Erdmann

#### Dermatoskopie

Für die unterschiedlichen Schweißdrüsenkarzinome existieren keine etablierten Diagnosekriterien der Dermatoskopie, die eine spezifische Diagnose und/oder eine Unterscheidung zwischen ihren benignen Pendants oder klinischen Differenzialdiagnosen ermöglichen. Anhand in einem Übersichtsartikel zusammengefasster Fallberichte weisen Schweißdrüsenkarzinome dermatoskopische Muster auf, die Basalzellkarzinome, kutane Plattenepithelkarzinome, melanozytäre Tumoren oder andere benigne Hauttumoren imitieren können.^98^

#### Optische Kohärenztomographie (OCT), Linefield konfokale optische Kohärenztomographie (LC‐OCT) und konfokale Reflektionsmikroskopie (RCM)

Für diese hochauflösenden Bildgebungsverfahren liegen bei Schweißdrüsenkarzinomen Fallberichte oder kleine Fallserien vor.

In der konfokalen Reflektionsmikroskopie (RCM) wies ein Porokarzinom‐Rezidiv Nester poroider Zellen neben Korrelaten duktaler Differenzierung mit umgebenden gewundenen, elongierten Gefäßen in dunklem Stromahintergrund auf.^99^

Beim extramammären Paget‐Karzinom zeigen sich in Fallserien in der RCM charakteristische Veränderungen mit histopathologischem Korrelat wie ein aufgehobenes Honigwabenmuster, intraepidermale Paget‐Zellen, basale ovoide Nester, Entzündungszellen und gewundene Gefäße.^100^ Zudem konnten mittels Linefield konfokaler optischer Kohärenztomographie (LC‐OCT) die einzelnen Paget‐Zellen mit dunklem Zytoplasma und hellem Zellkern von den umliegenden Keratinozyten abgegrenzt werden.^101^ Fallberichte mikrozystischer Adnexkarzinome zeigen in der OCT dermal runde, teils zwiebelschalenartig imponierende, zystische Areale neben linear angeordneten duktalen Strukturen und in der RCM Tumorinseln mit Spaltbildung und umgebender Fibrose.^102^, ^103^

#### Sonographie, Computertomographie (CT) und Magnetresonanztomographie (MRT)

Die Sonographie, Computertomographie (CT) oder Magnetresonanztomographie (MRT) liefern bei primären Schweißdrüsenkarzinomen im Regelfall keine zusätzlichen differenzialdiagnostischen Informationen, können jedoch dazu dienen, die Tiefeninfiltration präoperativ abzuschätzen sowie bei der Abgrenzung von histomorphologisch identischen Tumoren, wie Mammakarzinom‐ oder Adenokarzinom‐Metastasen hilfreich sein. Beim Primärstaging und der Nachsorge sind sie die Bildgebungsmethode der Wahl, insbesondere zur Beurteilung der regionären Lymphknotensituation und Ausschluss von Fernmetastasen.

## PROGNOSE UND STADIENEINTEILUNG

*Kapitelautor*: Carsten Weishaupt

Ab dem 01.01.2026 gilt die 9. Auflage des TNM‐Systems der UICC. Diese schließt nunmehr die Adnexkarzinome der Haut in die TNM Einteilung der „Karzinomen der Haut“ ein (Tabelle 2). Die Klassifikation für Tumoren in Kopf‐Hals‐Lokalisation unterscheidet sich davon nur in der N‐Einteilung (Tabelle 3).

**TABELLE 2:** TNM‐Klassifikation Hautkarzinome (ausgenommen Augenlider, Kopf‐Hals‐ Lokalisationen, Vulva, Penis und perianale Haut); die pT‐ und pN‐Kategorien entsprechen den cT‐ und cN‐Kategorien

T – Primärtumor	
cTX	Primärtumor kann nicht beurteilt werden
cT0	Kein Anhalt für Primärtumor
cTis	Carcinoma in situ
cT1	Tumor 2 cm oder weniger in größter Ausdehnung
cT2	Tumor mehr als 2 cm aber nicht mehr als 4 cm in größter Ausdehnung
cT3	Tumor mehr als 4 cm in größter Ausdehnung oder oberflächliche Knocheninvasion oder perineurale Invasion oder tiefe Invasion^*^
cT4a	Tumor mit makroskopischer Knocheninvasion / Knochenmarksinvasion
cT4b	Tumor mit Invasion des Achsenskeletts eingeschlossen Foramina und/oder Beteiligung des vertebralen Foramens bis zum Epiduralraum
^*^Eine “tiefe Invasion“ ist definiert als Invasion jenseits des subkutanen Fettgewebes oder > 6 mm (gemessen vom Stratum granulosum der benachbarten Epidermis bis zur Basis des Tumors). Eine perineurale Invasion ist definiert als Vorhandensein von Tumorzellen innerhalb einer Nervenscheide, die tiefer liegt als die Dermis oder 0,1 mm *oder* größer im Durchmesser misst *oder* als Infiltration von 5 oder mehr Nerven je Abschnitt. Im Falle multipler simultaner Tumoren wird der Tumor mit der höchsten T‐Kategorie klassifiziert und die Anzahl abgrenzbarer Tumoren in Klammern angegeben, z.B. T2(5).	

N ‐ Regionäre Lymphknoten	
cNX	Regionäre Lymphknoten können nicht beurteilt werden
cN0	Keine regionären Lymphknotenmetastasen
cN1	Metastase(n) in einem regionären Lymphknoten, 3 cm oder weniger in größter Ausdehnung
cN2	Metastase(n) in einem Lymphknoten, mehr als 3 cm, aber nicht mehr als 6 cm in größter Ausdehnung oder in multiplen Lymphknoten, keiner mehr als 6 cm in größter Ausdehnung

M – Fernmetastasen	
M0	Keine Fernmetastasen
M1	Fernmetastasen
Bilaterale oder kontralaterale Lymphknotenmetastasen (außer in Kopf‐Hals‐Lokalisation) werden als Fernmetatastasen klassifiziert.	

**TABELLE 3:** Klinische N‐Klassifikation Hautkarzinome in Kopf‐Hals‐Lokalisationen (ausgenommen Augenlider)

N‐Klassifikation	
cNX	Regionäre Lymphknoten können nicht beurteilt werden
cN0	Keine regionären Lymphknotenmetastasen
cN1	Metastase(n) in einem regionären Lymphknoten, 3 cm oder weniger in größter Ausdehnung
cN2a	Metastase(n) in solitärem ipsilateralem Lymphknoten, mehr als 3 cm, aber nicht mehr als 6 cm in größter Ausdehnung, ohne extranodale Ausbreitung
cN2b	Metastase(n) in multiplen ipsilateralen Lymphknoten, keine mehr als 6 cm in größter Ausdehnung, ohne extranodale Ausbreitung
cN2c	Metastase(n) in bilateralen oder kontralateralen Lymphknoten, keiner mehr als 6 cm in größter Ausdehnung, ohne extranodale Ausbreitung
cN3a	Metastase(n) in Lymphknoten, mehr als 6 cm in größter Ausdehnung, ohne extranodale Ausbreitung
cN3b	Metastase(n) in einem einzelnen oder multiplen Lymphknoten, klinisch mit extranodaler Ausbreitung
Eine Beteiligung der Haut oder der Weichteile (z.B. kutane/subkutane Satelliten‐ oder Intransit‐Metastasen) wird als „extranodale Ausbreitung“ (entsprechend N3b) gewertet.	
**Pathologische N‐Klassifikation Hautkarzinome in Kopf‐Hals‐Bereichen** (ausgenommen Augenlider)	
pNX	Regionäre Lymphknoten können nicht beurteilt werden
pN0	Keine regionären Lymphknotenmetastasen
pN1	Metastase(n) in einem regionären Lymphknoten, 3 cm oder weniger in größter Ausdehnung, ohne extranodale Ausbreitung
pN2a	Metastase(n) in solitärem ipsilateralem Lymphknoten, 3 cm oder weniger in größter Ausdehnung, mit extranodaler Ausbreitung oder mehr als 3 cm aber nicht mehr als 6 cm in größter Ausdehnung, ohne extranodale Ausbreitung
pN2b	Metastase(n) in multiplen ipsilateralen Lymphknoten, keine mehr als 6 cm in größter Ausdehnung, ohne extranodale Ausbreitung
pN2c	Metastase(n) in bilateralen oder kontralateralen Lymphknoten, keiner mehr als 6 cm in größter Ausdehnung, ohne extranodale Ausbreitung
pN3a	Metastase(n) in einem Lymphknoten, mehr als 6 cm in größter Ausdehnung, ohne extranodale Ausbreitung
pN3b	Metastase(n) in einem Lymphknoten mehr als 3 cm, mit extranodaler Ausbreitung oder in multiplen ipsilateralen Lymphknoten mit extranodaler Ausbreitung
Eine pathologisch extranodale Ausbreitung soll nur dann diagnostiziert werden, wenn ein Tumor, der sich vollständig innerhalb eines Lymphknotens erstreckt, durch die gesamte Dicke der Lymphknotenkapsel in das umgebende Bindegewebe eindringt, mit oder ohne Stromareaktion. Eine Weichteilablagerung an einer Stelle, an der ein Lymphknoten lokalisiert sein müsste, soll als mindestens ein Lymphknoten mit extranodaler Ausbreitung angesehen werden.	

Gesondert werden gemäß ihrer speziellen Lokalisation Tumoren der Augenlider, der Vulva, des Penis und der perianalen Haut klassifiziert. Für diese Lokalisationen wird auf die umfangreichen Klassifikationen der 9. Auflage der UICC verwiesen.

Eine Beteiligung der angrenzenden lokoregionalen Haut oder der Weichteile (z.B. kutane/subkutane Satelliten‐ oder Intransit‐Metastasen) wird nicht explizit erläutert. In der N‐Kategorie der Hautkarzinome in Kopf‐Hals‐Lokalisation wird eine solche als „extranodale“ Ausbreitung mit N3b bewertet (Tabelle 3, 4).

**TABELLE 4:** Stadien der Hautkarzinome einschließlich Kopf‐Hals‐Bereich (außer Augenlider)

**Stadium 0**	Tis	N0	M0
**Stadium I**	T1	N0	M0
**Stadium II**	T2	N0	M0
**Stadium III**	T3 T1, T2, T3	N0 N1	M0 M0
**Stadium IVA**	T1, T2, T3 T4	N2, N3 Jedes N	M0 M0
**Stadium IVB**	Jedes T	Jedes N	M1

In erster Linie sind die Schweißdrüsenkarzinome lokal fortschreitend.

Neben umweltbezogenen (Zugang zu spezialisierter medizinischer Versorgung) und sozioökonomischen Faktoren sind in der 9. TNM Auflage der UICC klinische und histopathologische Prognosefaktoren genannt, wobei diese systematisch für Schweißdrüsenkarzinome bislang nicht evaluiert sind.

Klinische Prognosefaktoren für Progress oder Rezidive umfassen:

• Das TNM Stadium (z.B. regionäre Lymphknotenmetastasen oder Fernmetastasen zum Zeitpunkt der Erstdiagnose)
• Lokalisation, Tumoren mit Ausdehnung auf die Orbita / Nebenhöhlen
• Immunsuppression
• Alter
• Rezidiverkrankung
• Wachstumsrate

Histologische Prognosefaktoren umfassen:

• Histopathologischer Typ
• Tumordicke
• Differenzierung
• Perineurale Invasion
• Lympho‐vaskuläre Invasion

Prinzipiell sind positive Schnittränder, große und dickere Tumoren, eine perineurale Invasion, hohe Mitoserate und / oder Lymphgefäßinvasion bei den meisten Entitäten Risikofaktoren für Lokalrezidive und / oder eine Metastasierung. Auf klinischer Seite spielen für aggressivere Verläufe vor allem eine Immunsuppression oder hämatoproliferative Begleiterkrankungen eine Rolle.

Tabelle 5 geht auf die in der Literatur beschriebene Prognose und die prognostischen Faktoren der Subtypen von Schweißdrüsenkarzinomen ein, die genannten Zahlen beruhen auf multiplen Fallserien und Kohorten.

**TABELLE 5:** Prognose und prognostische Faktoren.

Subtyp	Prognose	Prognostische Risiko‐Faktoren
Porokarzinom^20^, ^104^	5JÜR insg. 74,8 % 5JÜR St I+II 96 % 5J‐Lokalrezidivrate 11,5 % 5J‐Lokalrezidivrate St I+II: 20 % (Schnittränder 2‐3 cm) 31 % Metastasierung (58,5 % LK, 12,8 % Lunge; Leber, Gehirn möglich) 8,1 % Metastasierung bereits bei Diagnosestellung 67 % Mortalität bei LK‐Metastasierung, dann 1JÜR 84 %, 2JÜR 32 %	− 14 Mitosen/HPF − Tumoreindringtiefe > 7 mm − klarzellige Differenzierung − infiltratives und pagetoides Porokarzinom − lymphovaskuläre Invasion − hohes Alter
Hidradenokarzinom^36^	1JÜR 98,2 %; 3JÜR 97,2 %; 5JÜR 93,8 %; 10JÜR 90,5 % (alle tumorspezifisch) 2,4 %,Metastasierung (aktuelle Analysen), v.a. lymphatisch, zumeist innerhalb von zwei Jahren (2,1 % davon beriets bei Diagnosestellung (nach LK auch Lunge, Leber, Gehirn, Knochen)	Gesamtüberleben und tumorspezifisches Überleben v.a. abhängig von Tumorgröße
Maligner Mischtumor der Haut^105^	Lokalrezidivrate: 19,15 % (aufgrund kutaner Ausläufer) 60 % Metastasierung (LK, Lunge, Pleura, Gehirn, Knochen, Leber, Rückenmark, Niere, Schilddrüse) 19,15 % Metastasierung bei Diagnose 25 % Mortalität schlechtere Prognose für myoepitheliales Karzinom	
Spiradenokarzinom^32^, ^106^, ^107^	5JÜR 77 % Zeit bis Lokalrezidiv / Metastasierung: 20,1 Mo (0‐92 Mo) 24 Monate RFS: 37,2 % (45/121) 24 Monate Mortalitätsrate: 10,2 % (13/121) Medianes Überleben: 16 Monate Metastasierung 37,4 % (Lunge (34,7 %), Knochen (26,5 %), LK (53,1 %), Leber (14,3 %), Gehirn (10,2 %), Haut (10,2 %), Niere, Rückenmark, große Arterien und Becken jeweils (4,1 %), Mediastinum und Darm jeweils 2 % Lokalrezidive: 20,8 % Mortalität: 19,1 %	Brooke‐Spiegler Syndrom
Zylindrokarzinom^108^, ^109^	Metastasierung in 18/<50 Fällen in der Literatur beschrieben Mortalitätsrate: 35,5 % (11/31)	Brooke‐Spiegler‐Syndrom
Syringocystadenocarcinoma papilliferum^110^	8 % Rezidive (4/50) 20 % Metastasierung: LK, Lunge, Wirbelsäule, subkutan	Naevus sebaceus
)Extramammäres Paget‐Karzinom^111^, ^112^	Lokalrezidive 33‐40 % trotz ausgedehnter chirurgischer Maßnahmen Prognose v.a. abhängig von der Lokalisation und ob primäres oder sekundäres Paget‐Karzinom (Mortalität sekundärer extramammärer Paget‐Karzinom: 50 %) 5JÜR tumorspezifisch: 87 % (bei lokalem Rezidiv 92 %, bei regionaler Metastasierung 77 %, bei Fernmetastasierung 16 %) 1 LK Metastase → 5JÜR tumorspezifisch: 80 % ≥ 2 LK‐Metastasen → 5JÜR tumorspezifisch: 40 % Metastasierung: bis zu 17 %	− Tumoreindringtiefe > 1 mm − Alter > 65 Jahre − Lokalisation des Tumors (perianal schlechter als Rumpf oder Skrotum, vaginal schlechter als vulvär oder labial, Schleimhautbeteiligung → mehr LK‐Metastasen, Lokalrezidive) − R1‐Resektion → lokales Rezidivrisiko − lymphovaskuläre Invasion → Risiko für lokoregionäre und Fernmetastasierung − HER2+ → assoziiert mit hoher Tumoreindringtiefe und LK‐Metastasen
Mikrozystisches Adnexkarzinom^49^	Rezidivrate 4,3 % nach MKC (in der Regel in den ersten 2‐3 Jahren) 5J krankheitsspezifische Mortalität nach MKC 2 % Gesamtüberleben 10,8 Jahre Stadienabhängiges Gesamtüberleben: 5/10JÜR St I 86 %/77 %, St II 77 %/48 %, St III 65 %/44 % bei 68,5–74 % ist nur die Haut betroffen, bei 9–18,5 % sind hautumgebende Gewebe wie Unterhautfettgewebe, Muskel und Knochen betroffen; eine Metastasierung findet sich bei bis zu 1 % der Fälle	− Erkrankungsrisiko steigt mit dem Alter (mittleres Erkrankungsalter 63 Jahre SD ± 15,8) − Rezidivrisiko steigt um 11 % pro 1 cm^2^ Tumordurchmesser; Rezidivrisiko bei Tumordurchmesser ab 5,0 cm^2^ um das 13fache erhöht − perineurale Invasion − UV‐Exposition − vorangegangene Strahlentherapie − aggressive Verläuf sind bei Patienten mit Immunsuppression beschrieben
)Digitales papilläres Adenokarzinom^113^, ^114^	Lokalrezidivrate: 21‐50 % bei Erstdiagnose: 3–5 % LK‐Metastasen Metastasierung: 14–41 % (bis 20 Jahre nach Erstdiagnose), 83 % in regionale LK, 71 % Lunge	
Adenoid‐zystisches Karzinom^115^, ^116^	5JÜR 96,1 % Lokalrezidivrate: 44 % (bei inkompletter Exzision und bei perineuralem Wachstum; teilweise erst viele Jahre nach Erstdiagnose) 25 % regionale Metastasen 5 % Fernmetastasen (bis zu 18 Jahre nach Erstdiagnose), v.a. LK, Lunge, auch Brustwand, Perikard, Knochen, Nase und Leber bei Tumoren im äußeren Gehörgang und genital sowie bei perineuraler Ausbreitung höheres Risiko	Gering besseres Überleben für Männer, Patienten <50 Jahre, und Kopf‐Hals Tumoren
Muzinöses Karzinom der Haut^76^, ^77^ Endokrines muzinproduzierendes Schweißdrüsenkarzinom^79^	7 % Lokalrezidivrate nach Moh's Surgery/Schnellschnitten PFS: 36,5 Monate 10,5 % Metastasierung in regionale LK; 5,8 % in andere Organe, z.B. Lunge Lokalrezidivrate: 8,4–14,3 % Metastasierung 7,1 % (Parotis, LK, Speicheldrüsen, Lunge, Knochen, Pankreas)	Lokalisation am Stamm oder am Augenlid assoziiert mit schlechterem Verlauf nach Analyse von Fallberichten
)Kutanes adenosquamöses Karzinom^82^, ^83^, ^84^, ^117^	5JÜR < 30 % 25 % Lokalrezidivrate 13 % Metastasierung (LK, Lunge, Knochen)	
Kutanes (apokrines) kribriformes Karzinom^88^, ^118^, ^119^	Kein Rezidiv‐/Metastasierungsrisiko zwei Fälle mit persistierendem Befund nach unvollständiger Entfernung berichtet	
Sekretorisches Karzinom der Haut^90^, ^120^, ^121^ Mikrosekretorisches Karzinom der Haut^91^	Insgesamt indolent in einer Sammlung von 15 Fälle mit einer Nachbeobachtungszeit von bis zu 144 Monaten 1 pulmonale Metastasierung diagnostiziert Bislang 10 primär kutane Fälle beschrieben, darunter keine mit Rezidiven oder Metastasierung	
)Siegelringzell‐Karzinom^93^, ^94^	In der Literatur divergierende Angaben: Augenlidtumoren aggressives lokal‐infiltratives und metastasierendes Verhalten (42 % LK und Fernmetastasen) axilläre Tumoren seltener vor allem LK‐Metastasierung andere Fallserie: 19 % LK‐Metastasen bei Augelidtumoren vs. 72 % bei axillären Tumoren	
NUT‐Karzinom^97^	Bis 2025 17 Fälle mit primär kutanen NUT beschrieben: 5 Patienten entwickelten LK‐Metastasen; keine Fernmetastasen beschrieben	Extrakutane NUT haben eine sehr schlechte Prognose mit einem mittleren Überleben von 6,7 Monaten

Abk.: HPF, *high power field*; JÜR, Jahres‐Überlebensrate; LK, Lymphknoten; MKC, mikroskopisch kontrollierte Chirurgie; RFS, rückfallfreies Überleben

## THERAPIE

### Chirurgische Therapie

*Kapitelautoren*: Cornelia Erfurt‐Berge, Jochen Utikal

Die operative Therapie von Schweißdrüsenkarzinomen basiert in der Regel auf einer vollständigen chirurgischen Entfernung des Tumors. Für Schweißdrüsenkarzinome, generell wird die mikroskopisch kontrollierte Chirurgie (MKC) als Therapiestandard empfohlen, insbesondere für die periokulären Tumoren und andere chirurgisch schwierige Lokalisationen (Tabelle 6).^122^ Eine weite lokale Exzision (WLE) ist eine alternative Methode zur Entfernung der Tumoren mittels MKC – insbesondere bei Lokalisation außerhalb des Gesichts, wobei die Rezidivraten höher liegen.^123^ Für den Fall einer WLE ist ein Sicherheitsabstand von 1 cm sinnvoll. Periokuläre Tumoren bedürfen einer besonderen Aufmerksamkeit aufgrund ihrer funktionell‐anatomischen Besonderheiten.^80^, ^124^

**TABELLE 6:** Empfohlene Sicherheitsabstände bei Primärexzision und Lokalrezidiv.

	Sicherheitsabstand
Schweißdrüsenkarzinome allgemein	Mikroskopisch kontrollierte Chirurgie empfohlen; alternativ weite lokale Exzision mit Sicherheitsabstand von mind. 1 cm^*^
Extramammäres Paget‐Karzinom	Mikroskopisch kontrollierte Chirurgie empfohlen; alternativ präoperative Mapping‐Biopsien in Verbindung mit anschließender weiter lokaler Exzision
Mikrozystisches Adnexkarzinom	Mikroskopisch kontrollierte Chirurgie empfohlen; alternativ weite lokale Exzision mit Sicherheitsabstand von mind. 2 cm^*^
Digitales papilläres Adenokarzinom	Mikroskopisch kontrollierte Chirurgie empfohlen; normale Exzision oder Fingeramputation^*^

^*^ Mit deutlich höheren Rezidivraten

Generell gilt, dass das operative Vorgehen aufgrund einer unklaren Datenlage jeweils individuell je nach spezifischer Entität und Lokalisation festgelegt werden sollte.

Beim *extramammären Paget‐Karzinom* ist die Rückfallrate nach weiter lokaler Exzision mit 37,0 % sehr hoch.^125^ Durch große Sicherheitsabstände von 4 cm für den penoskrotalen und vulvären Bereich sowie 3,5 cm für perianale und axilläre Lokalisationen wird eine vollständige Tumorentfernung in 95 % der Fälle erreicht.^125^ Diese großen Sicherheitsabstände sind nicht immer anatomisch durchführbar. Daher werden Methoden zur Randkontrolle wie die MKC empfohlen.^126^ Die MKC einschließlich der mikrographischen Mohs‐Chirurgie erreicht eine niedrigere Rückfallrate von 18,7 %.^125^, ^127^ Die zirkumferentielle Beurteilung der peripheren und tiefen Ränder ist jedoch arbeitsintensiv und benötigt viele Gewebeschnitte. Aus diesem Grund wird die Durchführung präoperativer Mapping‐Biopsien (PMB) in Verbindung mit anschließender WLE empfohlen. Dieses Vorgehen führte zu einer 5‐Jahres‐Rückfallrate von 11,7 %, während WLE ohne PMB eine Rückfallrate von 45,5 % ergab.^126^

*Mikrozystische Adnexkarzinome* zeigen ein lokal aggressives Wachstumsverhalten und neigen häufig zu Rezidiven. Eine retrospektive Auswertung von Thomas et al. (2007) an 25 Patienten, die mittels MKC versorgt wurden, zeigte eine Rezidivrate von 12 %.^128^ Leibovitch et al. (2005) fanden in einer Analyse von 44 Fällen (davon 31,8 % Rezidive) in 17,5 % eine perineurale Invasion.^129^ Einer von 20 Patienten zeigte in einer 5‐Jahres‐Nachbeobachtung ein Rezidiv. Bezüglich des Sicherheitsabstandes gibt es derzeitig keine klare Evidenz. Es sollte möglichst immer eine MKC, alternativ eine WLE mit einem Sicherheitsabstand von 2 cm erfolgen.^130^

Das *digitale papilläre Adenokarzinom* neigt zu Lokalrezidiven. Daher ist eine sorgfältige chirurgische Sanierung die wichtigste therapeutische Maßnahme. Gemäß einer systematischen Übersichtsarbeit zeigten sich bei Einsatz einer MKC keine Rezidive, hingegen traten Rezidive nach normaler Exzision in 34,1 % und in 20 % nach Fingeramputation auf.^131^

Die verfügbaren Evidenzen zum Nutzen einer Sentinellymphknotenbiopsie (SLNB) bei Schweißdrüsenkarzinomen sind derzeit begrenzt. Insgesamt werden in frühen Tumorstadien nur selten postive SLN gefunde.^132^ Von insgesamt 41 Patienten mit Schweißdrüsenkarzinomen, die über einen Zeitraum von 19 Jahren diagnostiziert wurden, hatten 25 (61 %) eine SLNB erhalten. Von diesen hatte lediglich ein Patient eine SLN‐Beteiligung. In einer kleinen retrospektiven histopathologischen Untersuchung wurden sechs Fälle risikobehafteter (Einbruch in die Subkutis, im Primarius nachgewiesene intralymphatische Tumorzellen) Schweißdrüsenkarzinome (Hidradenokarzinom, ekkrines duktales Karzinom, Porokarzinom), bei denen eine SLNB durchgeführt wurde, re‐analysiert. Bei vier der sechs Fälle konnten Lymphknotenmetastasen des Schweißdrüsenkarzinoms bereits anhand der Hämatoxylin‐Eosin‐Färbung detektiert werden.^133^ Wie bei anderen Tumorarten scheint die Immunhistochemie die Erkennung kleiner mikroskopischer Metastasen im SLN zu erleichtern. Für eine SLNB als Stadieneinteilungsinstrument für Patienten Schweißdrüsenkarzinomen gibt es derzeit keine ausreichenden Daten. Bei Patienten mit positiven SLN oder einem klinischen Verdacht auf eine Lymphknotenmetastasierung kann eine Lymphknotendissektion in Erwägung gezogen werden. Die Entscheidung, ob eine Lymphknotendissektion durchgeführt wird, sollte individuell getroffen und im Rahmen einer interdisziplinären Tumorkonferenz besprochen werden.

### Strahlentherapie

*Kapitelautoren*: Clemens Seidel, Alexander Frisman

Eine Therapieentscheidung sollte unter Berücksichtigung der vorliegenden Klinik, patientenspezifischer Faktoren, (post‐)operativer Risikofaktoren und möglicher Kontraindikationen individuell und interdisziplinär im Rahmen einer Tumorboardbesprechung getroffen werden.

In Anbetracht der geringen Anzahl von prospektiven Studien ist eine Empfehlung zur Strahlentherapie basierend auf hohem Evidenzgrad oft schwierig. Die Literatur zu einzelnen Entitäten beschränkt sich oft auf wenige Fallserien oder retrospektive monozentrische Beobachtungen.^134^, ^135^, ^136^, ^137^, ^138^, ^139^

Bezüglich der spezifischen klinisch‐histologischen Entitäten finden in Analogie zu anderen Manifestationsorten nachfolgende Strahlentherapiekonzepte Anwendung.

*Extramammäres Paget‐Karzinom*: Eine Indikation zur postoperativen Strahlentherapie kann bei nichtinvasivem Tumor und großer Morbidität durch eine radikale Tumorentfernung oder Vorliegen von Risikofaktoren wie großem, rezidiviertem oder multifokalem Tumor, Resttumor (R1–R2), bei invasiven Tumoren, Lymphknotenmetastasen, positivem Schnittrand oder erhöhtem Ki67 % gestellt werden. Für makroskopische Tumoren wird eine Gesamtdosis (GD) von mehr als 60 Gy (EQD2) empfohlen. In der adjuvanten Situation finden im Tumorbettbereich 60 Gy und im Lymphabflussbereich 45–50 Gy GD (EQD2) Anwendung. In der aktuellen US‐amerikanischen Leitlinie für extramammäre Paget‐Karzinome sind Zielvolumina und Dosiskonzepte detailliert beschrieben.^125^

*Hidradenokarzinom*: Die Rückfallrate nach alleiniger Resektion liegt bei schlecht differenzierten (High‐Grade‐)Tumoren bis zu 50 %, entsprechend sollte anders als bei niedriggradigen (Low‐Grade‐)Tumoren, die nach R0‐Resektion eher selten Lokalrezidive zeigen, eine postoperative Bestrahlung erwogen werden.^35^, ^140^ Aufgrund der Radioresistenz von Hidradenokarzinomen wird eine Radiotherapie mit höherer Dosis empfohlen, in Abhängigkeit von der Lokalisation variieren diese zwischen 45 und 70 Gy GD.^141^

*Mikrozystisches Adnexkarzinom*: 2019 wurde eine kanadische Leitlinie für diese Entität publiziert.^130^ Eine primäre Strahlentherapie wird bei Inoperabilität empfohlen. Eine adjuvante Bestrahlung wird bei N1‐, R1‐ oder Rx‐Befund als indiziert angesehen. 60 Gy bis 66 Gy GD (EQD2) sind anzustreben.

*Porokarzinom*: Bei bestehenden Risikofaktoren sollte eine adjuvante Bestrahlung erwogen werden. In Fallserien wurden aufgrund systemischer Metastasierungsneigung die Lymphabstromgebiete meist inkludiert, sogar bei fehlendem Beweis eines Lymphknotenbefalls. Zur verwendeten GD liegen sehr heterogene Angaben von 24–70 Gy vor, meistens wurde aber die Dosis über 50 Gy (EQD2) verwendet. Damit ist eine Radiotherapie mit der GD von 50–70 Gy (EQD2) zu empfehlen.^141^

*Adenoid‐zystisches Karzinom*: In Analogie mit dem adenoid‐zystischen Karzinom der Speicheldrüsen in Kopf‐Hals‐Lokalisation wird bei makroskopischem Tumor eine GD von bis 70 Gy (EQD2) empfohlen. Für die adjuvante Situation wird eine Dosis mit 66 Gy bei R1‐Resektion und 50–60 Gy bei großen (pT3/pT4), R0‐resezierten Tumoren oder bestehender perineuraler oder lymphovaskulärer Invasion verwendet.

In palliativen, inoperablen Situationen oder bei symptomatischen metastatischen Manifestationen sind (in Analogie mit anderen Tumorentitäten) hypofraktionierte Dosiskonzepte mit Einzeldosen von 2,5–3,0 Gy bis zu einer GD von 36–45 Gy anwendbar.

Eine Verwendung von speziellen Techniken (zum Beispiel Brachytherapie) kann in individuellen Situationen mit potentiell großem therapeutischem Nutzen erwogen werden. Allerdings sollte in Anbetracht der geringen Evidenz über diese Therapie in einem interdisziplinären Tumorboardbesprechung individuell entschieden werden.

### Medikamentöse Therapie

*Kapitelautoren*: Ulrike Leiter, Alpaslan Tasdogan, Selma Ugurel

Zum Einsatz von Systemtherapien bei inoperablen oder metastasierten Schweißdrüsenkarzinomen gibt es aufgrund der Seltenheit dieser Tumorentitäten wenig Evidenz. Die Chemotherapie ist aktuell noch der Goldstandard für metastasierte Tumoren. Erstlinientherapie‐Protokolle umfassen Platinderivate, die in bis zu 60 % der Patienten eingesetzt werden, häufig in Kombination mit 5‐Fluorouracil (5‐FU) oder Capecitabin, während Cyclophosphamid, Doxorubicin, oder Vincristin zusammen mit Bleomycin Zweitlinientherapien darstellen.^13^ Eine Übersichtsarbeit von Mijamoto et al. befasst sich mit 28 Patienten, die aufgrund einer fortgeschrittenen ekkrinen Porokarzinomerkrankung eine Chemotherapie erhalten haben.^118^ Es zeigte sich eine objektive Ansprechrate von 31,3 %, was für eine eher mäßige bis geringe Chemosensitivität dieser Tumoren spricht. Eine aktuelle systematische Übersichtsarbeit und Metaanalyse von Abdullah et al., die die Primärversorgung von mehr als 1000 Patienten mit Porokarzinom umfasst, ergab nur für einen geringen Anteil von < 1 % der Patienten eine Notwendigkeit des Einsatzes einer Systemtherapie.^142^ Diese stellte in allen Fällen eine Chemotherapie dar, entweder in Kombination mit einer Operation oder mit einer Strahlentherapie.

Zielgerichtete Therapien wie Trastuzumab wurden zur Krankheitsstabilisierung bei Tumoren mit HER2/neu Expression eingesetzt.^13^ Einzelne Fallberichte existieren zum Einsatz von EGFR‐Inhibitoren bei metastasiertem Schweißdrüsenkarzinom, insbesondere Cetuximab.^7^, ^20^, ^143^, ^144^ Teilweise kam dieses in Kombination mit Paclitaxel zum Einsatz, hierunter zeigte sich in einem Fall ein komplettes Ansprechen der Fernmetastasen über eine Nachbeobachtungszeit von 6 Monaten.^144^ Eine Studie von Denisova et al. zeigte, dass Mutationen im *TP53*‐Gen sowie genetische Alterationen in HRR‐assoziierten Genen (hoher HRD‐Score) bei Porokarzinomen häufig auftreten (in circa 50 % der Fälle), so dass beispielsweise PARP‐Inhibitoren eine potenziell vielversprechende zielgerichtete Therapieoption darstellen könnten.^6^

Die Immuncheckpointblockade mit PD‐1‐Antikörpern stellt ebenfalls eine mögliche Therapieoption dar. Eine PD‐L1‐Expression konnte in 13 % der Schweißdrüsenkarzinome nachgewiesen werden.^16^ Es wurden Einzelfälle mit partiellem oder komplettem Ansprechen in Zweit‐ oder weiteren Folgetherapien über 18 beziehungsweise 22 Monate beschrieben.^145^, ^146^, ^147^, ^148^ Zudem existiert ein Fallbericht mit einer über 12 Monate anhaltenden partiellen Remission unter Erstlinientherapie mit Pembrolizumab.^149^ Ein besseres Ansprechen könnte bei UV‐assoziierten Tumoren vorliegen.

Intraläsionale Therapieoptionen können ebenfalls in Betracht gezogen werden. So beschreibt ein Fallbericht eine komplette Remission eines metastasierten Porokarzinoms nach intraläsionaler Gabe von Interferon‐alpha plus Interleukin‐2.^150^

Nach aktueller Datenlage sind die Erfolgsaussichten einer Systemtherapie beim Schweißdrüsenkarzinom limitiert. Eine Bestimmung der Tumormutationslast im Gewebe, des Vorliegens einer UV‐Signatur, der PD‐L1‐Expression im Tumor, sowie die Suche nach Treibermutationen, die Angriffspunkte einer individualisierten zielgerichteten Therapie darstellen könnten, erscheint daher sinnvoll.^6^

## NACHSORGE

*Kapitelautor*: Ulrike Leiter

Bisher gibt es in der Literatur nur wenige Untersuchungen zur Nachsorge von Schweißdrüsenkarzinomen. Hidradenokarzinome, Porokarzinome, digitale papilläre Adenokarzinome sowie Pilomatrixkarzinome zeigen neben häufigen Rezidiven auch eine Metastasierungsneigung.^27^, ^151^, ^152^, ^153^ Da mehr als 60 % aller metastasierenden Patienten innerhalb von 2 Jahren sowohl lymphogene als auch primär hämatogene Rezidive entwickeln, werden in den ersten zwei Jahren nach Erstdiagnose Nachsorgeintervalle zwischen 3 und 6 Monaten empfohlen.^151^, ^154^ Anschließend werden bis 5 Jahre nach Erstdiagnose sechsmonatliche oder jährliche Kontrollen angestrebt (Tabelle 7).^130^

**TABELLE 7:** Nachsorgeschema für Schweißdrüsenkarzinome (in Anlehnung an das Mannheimer Nachsorgeschema für maligne Adnextumoren der Haut, modifiziert nach dem Nachsorgeschema für Patienten mit kutanen Hochrisiko‐Plattenepithelkarzinomen der S3‐Leitlinie).^157^

	Klinische Untersuchung^*^		Sonographie Narbe, Intransit‐ und regionale Lymphknoten		
	*Jahr 1–2*	*Jahr 3–5*	*Jahr 1–2*	*Jahr 3–5* ^***^	Schnittbildgebung
Ersttumor	2 x	1 x	–	–	
Rezidiv‐Tumor	4 x	2 x	2 x	1 x	Bei Verdacht auf Metastasierung oder fortgeschrittene Tumoren
Tumoren mit lokoregionärer, Lymphknoten‐ oder Fernmetastasierung	4x	4 x	2–4 x	1–2 x	Individuell^**^ Bei Verdacht auf Metastasierung oder fortgeschrittene Tumoren

* Anamnese, Untersuchung des kompletten Integuments, Inspektion und Palpation der Lymphknotenstationen und Lymphabflusswege.

** Bei Verdacht auf Metastasierung oder lokal fortgeschrittenen Tumoren zur Abklärung tiefer Infiltration sowie bei Verdacht auf perineurales Wachstum.

*** Ausnahme ist das digitale papilläre Adenokarzinom, wo die Nachsorge bis zu 10 Jahren empfohlen wird.

Die Nachsorge sollte eine Untersuchung des gesamten Integuments inklusive Palpation der Lymphknotenstationen und ‐abflusswege enthalten. Eine Anleitung zur Selbstuntersuchung ist ebenfalls empfehlenswert.^155^ Da es wenige Daten bezüglich eines Nachsorgeschemas bei Adnextumoren gibt, wird eine Nachsorge nach dem Mannheimer Schema, in Anlehnung an das Nachsorgeschema für Hochrisikoplattenepithelkarzinome der Haut gemäß der S3‐Leitlinie, empfohlen. Die Nachsorgeintervalle sowie der Einsatz bildgebender Maßnahmen können individuell und risikoadaptiert angepasst werden. Außerdem ist eine Anleitung für Patienten zur Selbstuntersuchung ratsam. Regelmäßige Ultraschalluntersuchungen werden vor allem für die Nachsorge von Patienten mit erhöhtem Rezidiv‐ und Metastasierungsrisiko empfohlen.^20^, ^156^ Eine Schnittbildgebung mittels CT, PET‐CT oder MRT kann nach klinischer Indikation und in Abhängigkeit des Krankheitsstadiums erfolgen. Aufgrund der Seltenheit dieser Tumoren und der potenziell aggressiven und letalen Verläufe ist eine Anbindung an ein Hauttumorzentrum erstrebenswert.

## VERFAHREN DER KONSENSBILDUNG

Die aktualisierte Fassung der Leitlinie wurde erstellt im Auftrag der *Arbeitsgemeinschaft Dermatologische Onkologie der Deutschen Krebsgesellschaft* und der *Deutschen Dermatologischen Gesellschaft*.

Die wichtigsten Empfehlungen dieser Leitlinie werden in Tabelle 8 zusammengefasst. Im Rahmen des Managements der Interessenkonflikte: Experten mit einem möglichen Interessenkonflikt haben an der Formulierung von Empfehlungen zu entsprechenden Themen nicht mitgewirkt. Die Bewertung der Interessenskonflikte der einzelnen Experten erfolgte durch die Leitlinienkoordinatoren; die Leitlinienkoordinatoren wurden durch die Leitlinienbeauftragte der ADO/DKG beurteilt.

**TABELLE 8:** Übersicht über die wichtigsten Aussagen und Empfehlungen der S1‐Leitlinie zu Schweißdrüsenkarzinomen (Stand 2025).

Thematik	Aussage/Empfehlung nach Leitlinie
Epidemiologie	Sehr seltene Entitäten; < 0,05 % aller malignen Hauttumoren; Nach dem Basalzellkarzinom häufigste Gruppe innerhalb der Adnexkarzinome; Steigende Inzidenz; Frauen und Männer bis auf wenige Entitäten (wie Siegelringzellkarznom der Haut) weitgehend gleichermaßen häufig betroffen; Erstmanifestation häufiger im fortgeschrittenen Lebensalter.
Ätiopathogenese	Entstehung meist *de‐novo*, selten auf dem Boden benigner Pendants; Risikofaktoren: ultraviolette Strahlung, Strahlentherapie, Immunsuppression, humane Papillomviren, genetische Aberrationen, genetische Syndrome (Brooke‐Spiegler‐Syndrom), hämatoproliferative Grunderkrankungen
Klassifikation	Unterteilung in mehr als 20 Entitäten; Einteilung in ekkrine oder apokrine Karzinome nicht immer möglich und prognostisch‐therapeutisch nicht relevant; Klinisch‐pathologische TNM‐Klassifikation gemäß 9. Auflage der UICC Klassifikation, gültig seit 01.01.2026; Die wichtigsten Entitäten: Porokarzinom, Hidradenokarzinom, maligner Mischtumor der Haut, Spiradenokarzinom / Zylindrokarzinom (häufig beim Brooke‐Spiegler‐Syndrom), Syringocystadenocarcinoma papilliferum, extramammäres Paget‐Karzinom (beachte hier Fallstrick entzündliche Dermatose), mikrozystisches Adnexkarzinom (typischerweise Wangen), digitales papilläres Adenokarzinom (typischerweise an Fingern/Zehen), adenoid‐zystisches Karzinom, muzinöses Karzinom
Klinik	Unspezifische Klinik. Exzision meist unter Verdachtsdiagnose gutartige Zyste, Basalzellkarzinom, Plattenepithelkarzinom; Meist Kopf‐Hals‐Lokalisationen
Diagnostik	Probeexzision / Exzision zur diagnostischen Sicherung; Histopathologische Kriterien: apokrine oder ekkrine Differenzierung, Zellatypien, Kernpleomorphien, dicht gedrängt stehende Zellkerne („Crowding“), zahlreiche Mitosen, infiltrierendes Wachstum; Immunhistochemische Marker: u. a. Zytokeratine (CK) 7, Gross cystic disease fluid protein 15 (GCDFP‐15), Carcinoembryonales Antigen (CEA), epitheliales Membranantigen (EMA), nuclear protein of testis (NUT); keine etablierten Diagnosekriterien der Dermatoskopie oder anderer nichtinvasiver In‐vivo‐Diagnostik; Bildgebung bei Risikofaktoren im Primärstaging und evtl. in der Nachsorge: lokoregionäre Sonographie; Computertomographie/Magnetresonanztomographie zur Einschätzung der Primärtumorausdehnung und Ausschluss einer Metastasierung; Wächterlymphknotenbiopsie (SLNB): keine allgemeinen Empfehlungen, Fall‐zu‐Fall Entscheidung basierend auf den klinisch‐histopathologischen Risikofaktoren
Prognostische Faktoren	Aufgrund der Seltenheit existiert keine eigene Stadieneinteilung; eine Einteilung in ein lokales, ein lymphonodal metastasiertes, und ein fernmetastasiertes Stadium ist sinnvoll; Eine Metastasierung erfolgt zunächst lokal fortschreitend (per continuitatem), später mit Befall der regionären Lymphknoten und mit Fernmetastasen (meist Lunge); Risikofaktoren für Lokalrezidiv / Metastasierung: positive Schnittränder, große und dickere Tumoren, perineurale Invasion, hohe Mitoserate, Lymphgefäßinvasion
Chirurgische Therapie	Das operative Vorgehen sollte individuell nach Entität und Lokalisation festgelegt werden; Therapie der ersten Wahl: vollständige chirurgische Exzision unter allseitiger histologischer Schnittrandkontrolle (mikroskopisch kontrollierte Chirurgie, MKC); Alternativ: weite lokale Exzision (WLE) mit Sicherheitsabstand von mindestens 1 cm, beim mikrozystischen Adnexkarzinom 2 cm (hohe Lokalrezidivrate); Beim extramammären Paget‐Karzinom Empfehlung zur Durchführung präoperativer Mapping‐Biopsien zur Bestimmung der Tumorausdehnung
Strahlentherapie	Keine prospektiven Studien; Datenlage basiert auf Fallserien oder retrospektiven monozentrischen Beobachtungen; Die Empfehlung zur Strahlentherapie sollte individuell im interdisziplinären Tumorboard erfolgen; Indikationen zur Strahlentherapie: voraussichtliche hohe Morbidität durch radikale Tumorchirurgie, ausgedehnter / rezidivierter / multifokaler Tumor, Resttumor (R1/R2), Lymphknotenmetastasen; Eine postoperative adjuvante Strahlentherapie des Tumorbetts, ggfs. unter Einbezug der regionären Lymphknotenstation, ist für die meisten Tumoren eine fallbasietre interdisziplinäre Entscheidung unter Berücksichtigung von Risikofaktoren
Medikamentöse Therapie	Keine prospektiven Studien; Datenlage basiert auf Fallserien oder retrospektiven monozentrischen Beobachtungen; Die Empfehlung zur medikamentösen Therapie sollte individuell im interdisziplinären Tumorboard erfolgen; Chemotherapie ist Therapie der ersten Wahl, Kombinationschemotherapie mit Platin empfohlen, Ansprechrate circa 30 %; Zielgerichtete Therapien möglich, zum Beispiel mit HER2‐Inhibitoren (bei Expression) oder EGFR‐Inhibitoren (bei Überexpression), ggfs. in Kombination mit Chemotherapie; Für Immuncheckpoint‐Inhibitoren (PD‐1) wurde nur in Einzelfällen ein Therapieansprechen berichtet
Nachsorge	Keine systematischen Untersuchungen; Es wird eine Nachsorge in Anlehnung an das Nachsorgeschema für Hochrisikoplattenepithelkarzinome der Haut (S3‐Leitlinie) empfohlen, unter Einbezug einer klinischen Untersuchung, Sonographie, sowie ggfs. Schnittbildgebung

*Leitlinienkoordinatoren*: Prof. Dr. med. Mirjana Ziemer, Leipzig; PD Dr. med. Michael Erdmann, Erlangen

*Leitlinienbeauftragte der ADO/DKG*: Prof. Dr. med. Selma Ugurel, Bielefeld

## DANKSAGUNG

Open access Veröffentlichung ermöglicht und organisiert durch Projekt DEAL.

## INTERESSENKONFLIKT

A.A., A.B.‐A., D.H., C.E.‐B., A.F., C.H., T.M., L.R., S.R., S.S., C.S. deklarieren keine Interessenskonflikte.

F.M. hat Reisekostenzuschüsse und/oder Vortragshonorare und/oder Beraterhonorare von Novartis, Roche, BMS, MSD, Pierre Fabre, Sanofi, Immunocore und Regeneron erhalten, jeweils außerhalb der eingereichten Arbeit.

J.U. hat im Rahmen von Advisory Board‐Tätigkeiten oder von Vortragstätigkeiten von folgenden Firmen Honorare oder Reisekosten erhalten: Amgen, BMS, GSK, Immunocore, Leo Pharma, MSD, Novartis, Pierre Fabre, Rheacell, Roche und Sanofi, jeweils außerhalb der eingereichten Arbeit.

S.U. erhielt Forschungsförderung durch BMS, Merck; Vortragshonorare von BMS, Merck, MSD, Regeneron; Beraterhonorare von BMS, Menarini Stemline, Merck, MSD, Regeneron; sowie Unterstützung für Reisenkosten und Gebühren zu Tagungen von Almirall, BMS, IGEA, MSD, Pierre Fabre, Sun Pharma. jeweils außerhalb der eingereichten Arbeit.
